# Synthesis of new allylidene amino phenol-containing Schiff bases and metal complex formation using trimethinium salts†

**DOI:** 10.1039/d1ra04214a

**Published:** 2021-06-18

**Authors:** Ziba Rafiee Samani, Abdolmohammad Mehranpour

**Affiliations:** Department of Chemistry, Faculty of Sciences, Persian Gulf University Bushehr 75169 Iran ammehranpour@hotmail.com

## Abstract

An efficient route for the synthesis of novel Schiff bases from the condensation reaction of 2-substituted 1,3-bis(dimethylamino)-trimethinium salts with diverse aminophenols in the presence of triethylamine in EtOH at reflux is described. Complexes of transition metals with Schiff base ligand (L) 3c, having the donor atom set N_2_O_2_, were studied. The ultraviolet spectral behavior of the complexes in DMSO was investigated and the *λ*_max_ of these compounds was examined. The structure of the new compounds was confirmed based on their spectral data from IR, ^1^H NMR and ^13^C NMR, mass spectra, and elemental analysis.

## Abstract 2

An efficient route for the synthesis of novel Schiff bases from the condensation reaction of 2-substituted 1,3-bis(dimethylamino)-trimethinium salts with diverse aminophenols in the presence of triethylamine in EtOH at reflux is described.

## Graphical abstract

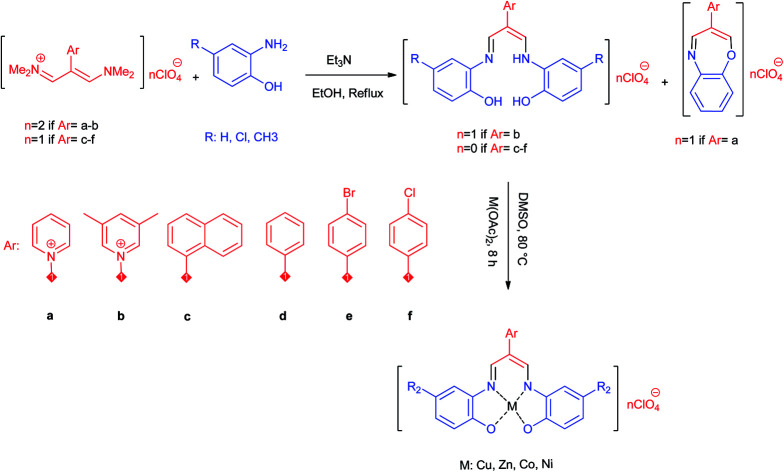

## Introduction

Schiff bases are some organic compounds that are highly used. They are used as pigments and dyes, intermediates in organic synthesis, catalysts, and polymer stabilizers. Schiff bases exhibit a wide range of biological activities, including antifungal, antiviral, antimalarial, antibacterial, anti-inflammatory, antiproliferative, and antipyretic properties.^1–6^

Schiff base ligands have the capability of coordinating metals through imine nitrogen and another group. Nowadays active and well-designed Schiff base ligands are considered “privileged ligands”. Schiff bases are able to stabilize many different metals in various oxidation states, controlling the performance of metals in a wide variety of useful catalytic transformations.^7–9^

Metal complexes of Schiff bases play a central role in the development of coordination chemistry. This situation is manifested by the huge number of publications ranging from purely synthetic to modern physicochemical to biochemically relevant studies of these complexes. A wide variety of stable chemical species have been synthesized containing both transition and nontransition metals and multifarious ligand systems.^10,11^

The vinamidines, with saturated nitrogen as the p-donor and the imino group as the π-acceptor, are of particular interest to us. Vinamidinium salts have long found practical use as versatile three-carbon building blocks in the synthesis of heterocyclic benzenoid and nonbenzenoid aromatic rings, from cyclic and acyclic precursors alike. One of the useful attributes of many vinamidinium salts is their ease of preparation from substituted acetic acids under Vilsmeier–Haack conditions.^12–23^ “In this study, our research group was able to prepare β-substituted trimethinium salts with different R groups (including aryls and heteroaryls) from correspondingly substituted acetic acids, R–CH_2_CO_2_H, with good yields”.^24,25^

**Scheme 1:**
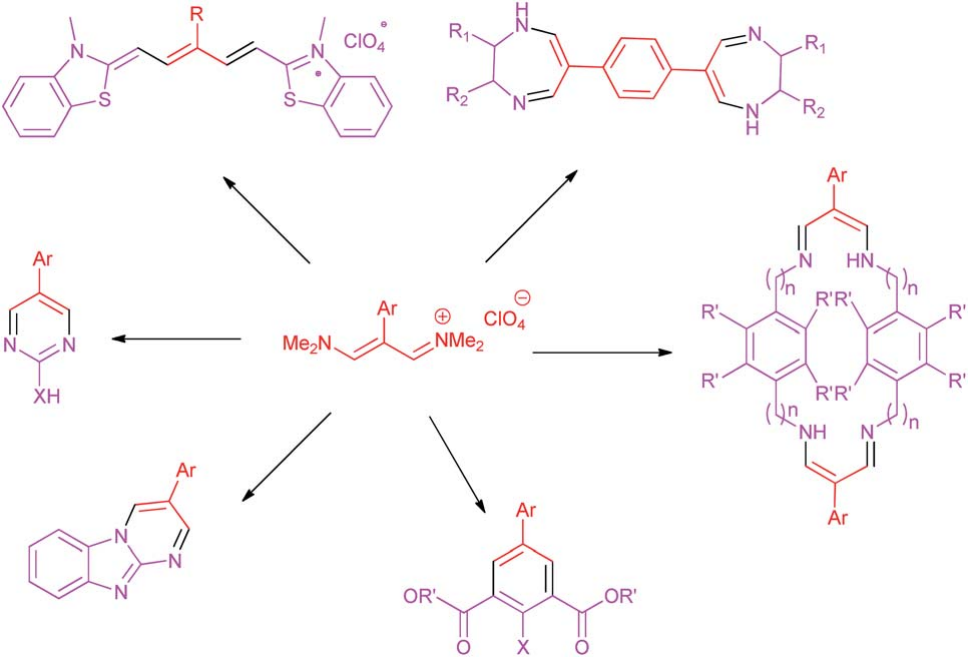
Application of trimethinium salts in synthetic organic chemistry.

**Scheme 2:**
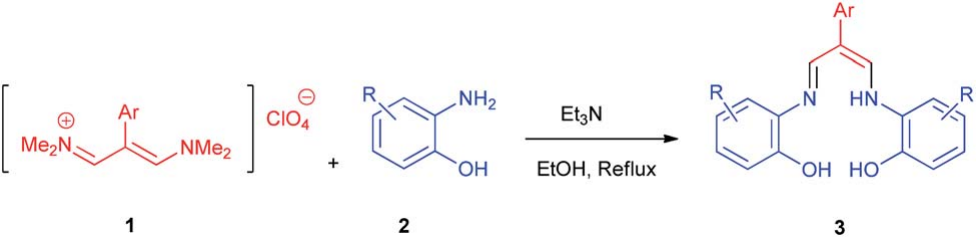
Synthesis of Schiff bases 3*via* the reaction between trimethinium salts 1 and diverse aminophenols 2 in the presence of Et_3_N in EtOH at reflux.

In continuation of this research on applications of trimethinium salts in organic synthesis (Scheme 1),^26–31^ in this study, we report a new and highly efficient method for the synthesis of a novel class of allylidene amino phenol-containing Schiff bases from the reaction of trimethinium salts and diverse aminophenols in the presence of triethylamine, under catalyst-free conditions in EtOH at reflux (Scheme 2).

## Results and discussion

Eight new Schiff base derivatives with the general formula N_2_O_2_, were synthesized from the reaction of different aminophenols and various trimethinium salts in the presence of triethylamine in ethanol as a solvent at reflux. Initially, *N*-(2-(4-bromophenyl)-3-(dimethylamino)-allylidene)-*N*-methylmethanaminium perchlorate (1e) and 2-amino-4-chlorophenol (2b) were chosen as the model substrates to optimize the reaction conditions, (the reason for this choice is the good TLC of these two reactants for observations), such as various reagent sources and solvents. The results are summarized in Table 1. In this study, various reagents such as NaH and NaOCH_3_ were examined and it became clear that they cannot continue this reaction (Table 1, entries 1 and 2). The bases such as Et_3_N and i-Pr_2_NEt were examined in EtOH, in which resulted higher yield and shorter reaction time when the reaction was carried out in the presence of (1 eq.) of the Et_3_N (Table 1, entries 3 and 4). The effect of solvents were also investigated and it was observed that the desired product was not obtained in the solvents CH_3_CN and DMF. However, the reaction was obtained highly effective with solvents such as EtOH and MeOH (Table 1, entries 4–7). The control experiment confirmed that the reaction has not occur in the absence of the base and acid conditions (Table 1, entries 8 and 9).

**Table 1:** Optimization of the reaction conditions^a^

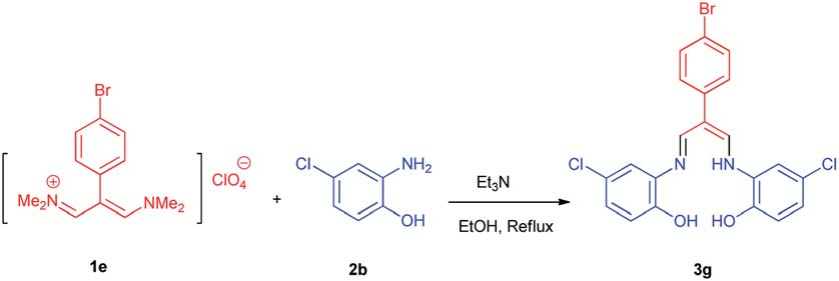				
Entry	Conditions	Solvent	Time (h)	Yield^b^ (%)
1	NaH	EtOH	24	—
2	NaOCH_3_	EtOH	24	—
3	i-Pr_2_NEt	EtOH	20	55
4	Et_3_N	EtOH	12	90
5	Et_3_N	MeOH	12	70
6	Et_3_N	CH_3_CN	24	—
7	Et_3_N	DMF	24	—
8	—	EtOH	24	—
9	AcOH	EtOH	24	—

a Reaction conditions: *N*-(2-(4-bromophenyl)-3-(dimethylamino)-allylidene)-*N*-methylmethanaminium perchlorate 1e (1 mmol), 2-amino-4-chlorophenol 2b (2 mmol), base (1 eq.), solvent (15 mL), 12 h.

b Isolated yield.

In the next stage, the efficiency of the process under optimized conditions was explored. For this purpose, trimethinium salts 1a–f were condensed with aminophenol derivatives 2a–c in the presence of Et_3_N (1 eq.) to afford the corresponding products 3a–i in high yields.

The synthetic pathway to synthesis the titled compounds is consisting of two steps. At first, compounds 1 were prepared similar to the previous studies.^18–25^ Then the results were treated with aminophenol derivatives to afford the related Schiff base ligands as the desired products. As Table 2 indicates, a variety of trimethinium salts were successfully applied in this process to afford the corresponding Schiff base ligands derivatives as novel compounds with excellent yields.

**Table 2:** Synthesis of product 3*via* the reaction of 2-substituted trimethinium salts 1 with aminophenol derivatives 2 in the presence of Et_3_N in ethanol at reflux

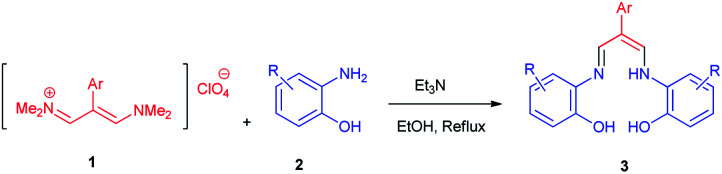					
Entry	Trimethinium salts 1	R	Product 3	Time (h)	Yield^a^ (%)
1	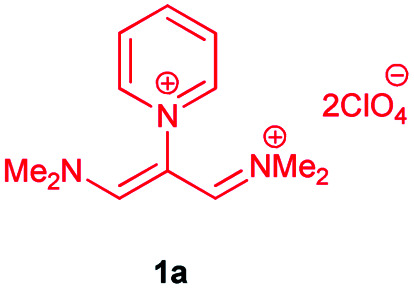	H	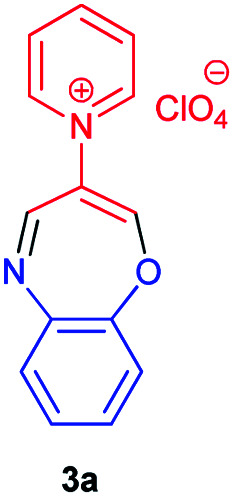	14	90
2	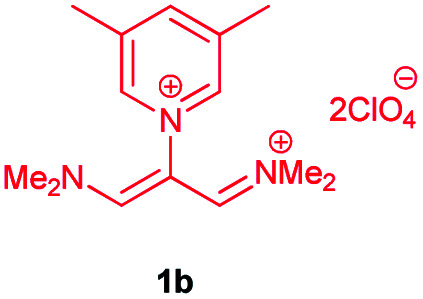	**H**	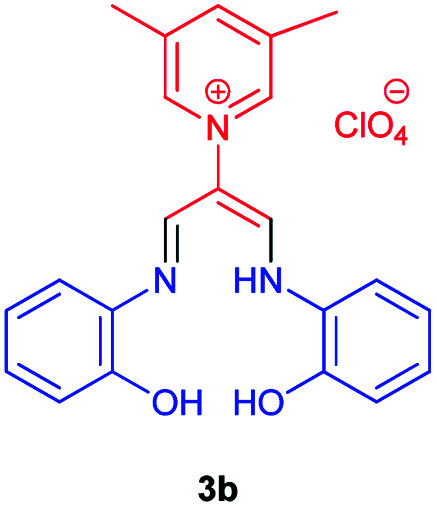	10	88
3	1b	CH_3_	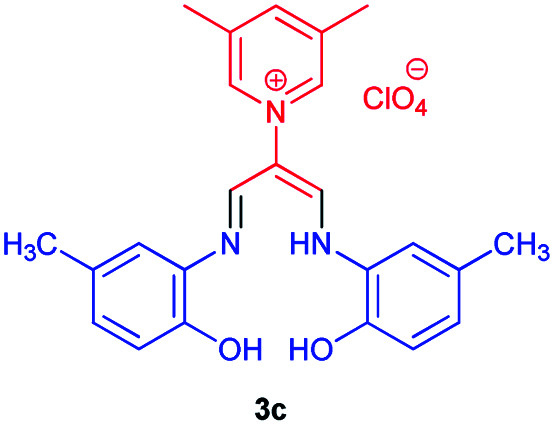	10	98
4	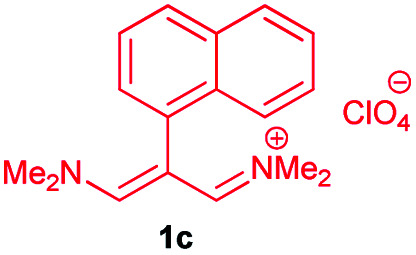	Cl	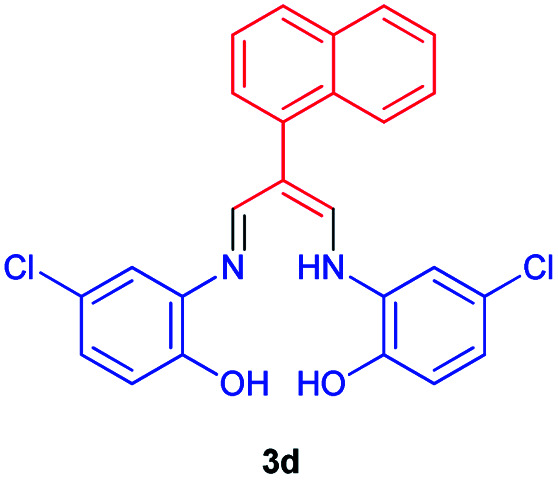	15	88
5	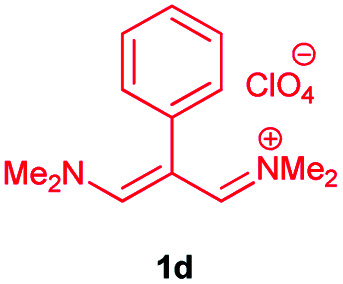	**CH**_**3**_	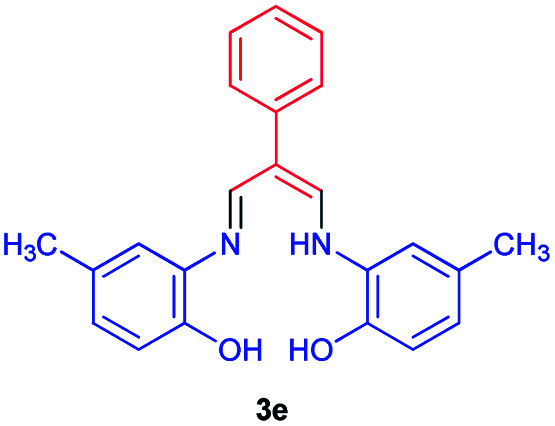	12	60
6	1d	**Cl**	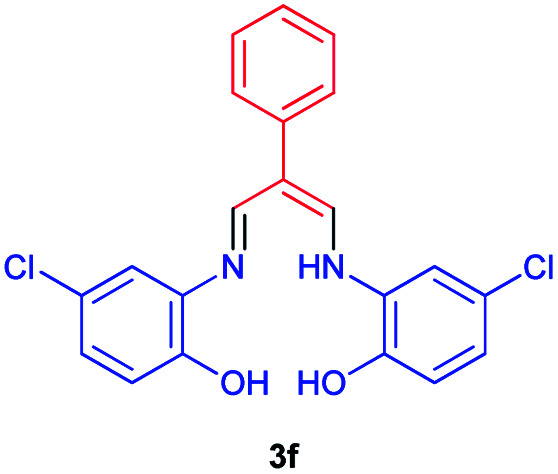	14	90
7	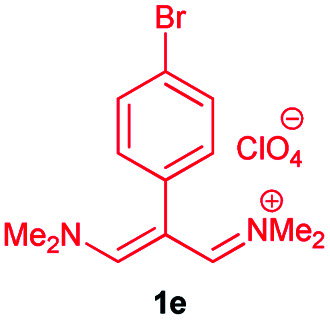	**Cl**	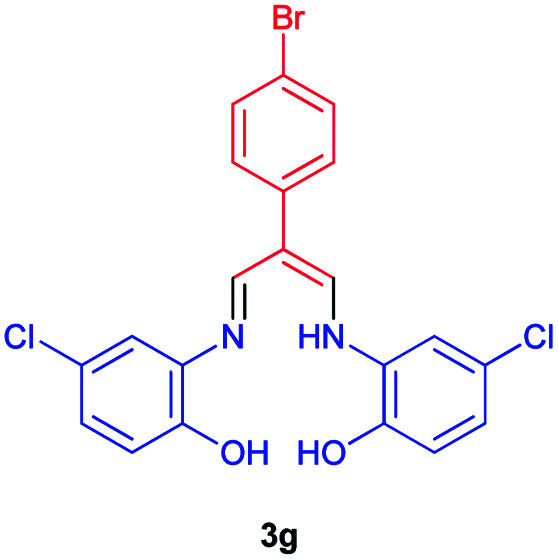	12	95
8	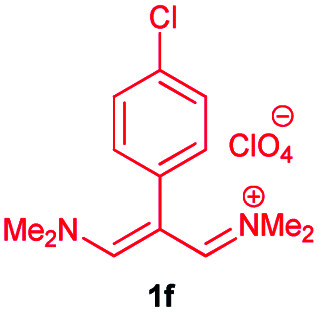	**Cl**	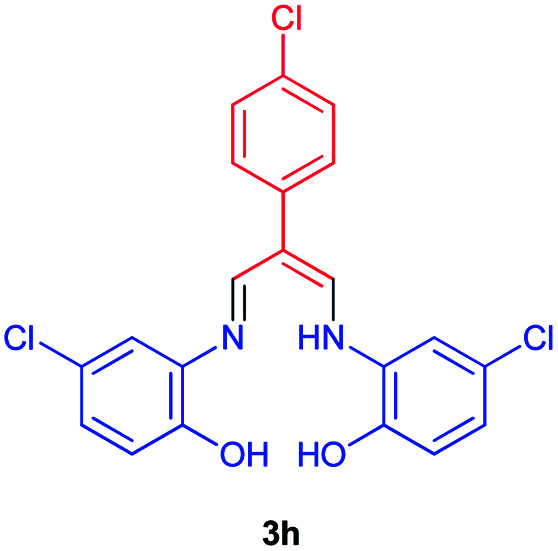	14	93
9	1f	**H**	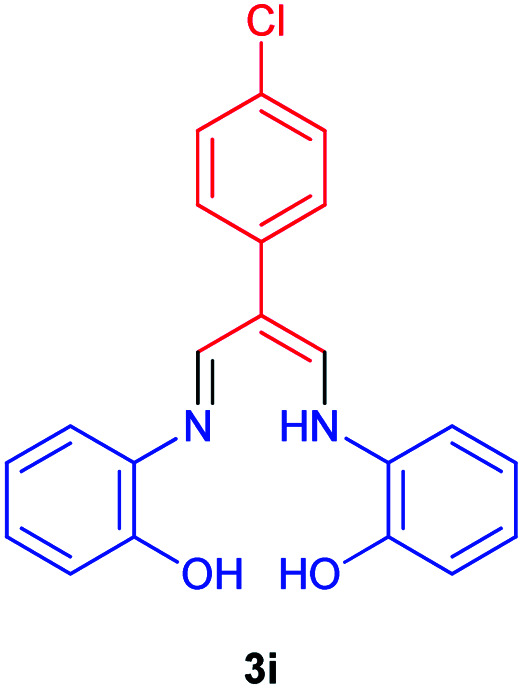	14	90

a Isolated yield.

As shown in Table 2, compound 3a unlike the other compounds, creates the 1,4-oxazpine salt as a product, probably due to the lack of electrons of the unsubstituted pyridine ring, which causes an intramolecular reaction. In the other molecules, the electron deficiency is less and there is enough time to perform the intermolecular reaction.

### The complexation steps

**Scheme 3:**
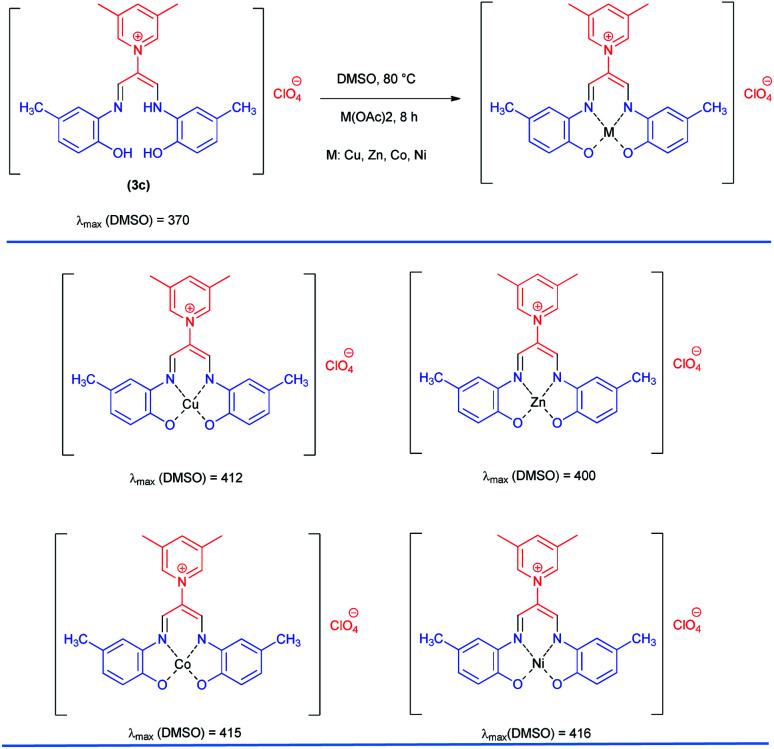
Preparation of Schiff base complexes^*a*^. ^*a*^Reaction conditions: 1-((2-hydroxy-5-methylphenyl)amino)-3-(((2-hydroxy-5-methylphenyl)imino)prop-1-en-2-yl)-3,5-dimethylpyridinium perchlorate 3c (1 mmol), M(OAc)_2_ (M ∼ Cu, Zn Co, Ni) (1 mmol), DMSO (3 mL) at 100 °C for 6 h.

The complexation of a series of new allylidene amino *ortho* hydroxy-containing Schiff bases as ligand with different metal ions was studied by means of the ultraviolet-visible spectrophotometry technique in DMSO as a solvent. Copper, zinc, cobalt, and nickel Schiff bases were prepared using the corresponding acetate M(OAc)_2_ (M ∼ Cu, Zn, Co, Ni). For this purpose, compound 3c was chosen; then, UV-Vis absorption spectrum of the ligand 3c and its complexes were investigated in range of 190–840 nm in DMSO solvent (Scheme 3). The ultraviolet-visible electronic spectrum of ligand 3c shows the absorption peak at (370) nm, which can be attributed to n → π* electronic transitions. As shown in Scheme 3, for all complexes, maximum absorption wavelengths shifted to longer values (bathochromic effect) compared to the ligand 3c indicating the complex formation.

**Scheme 4:**
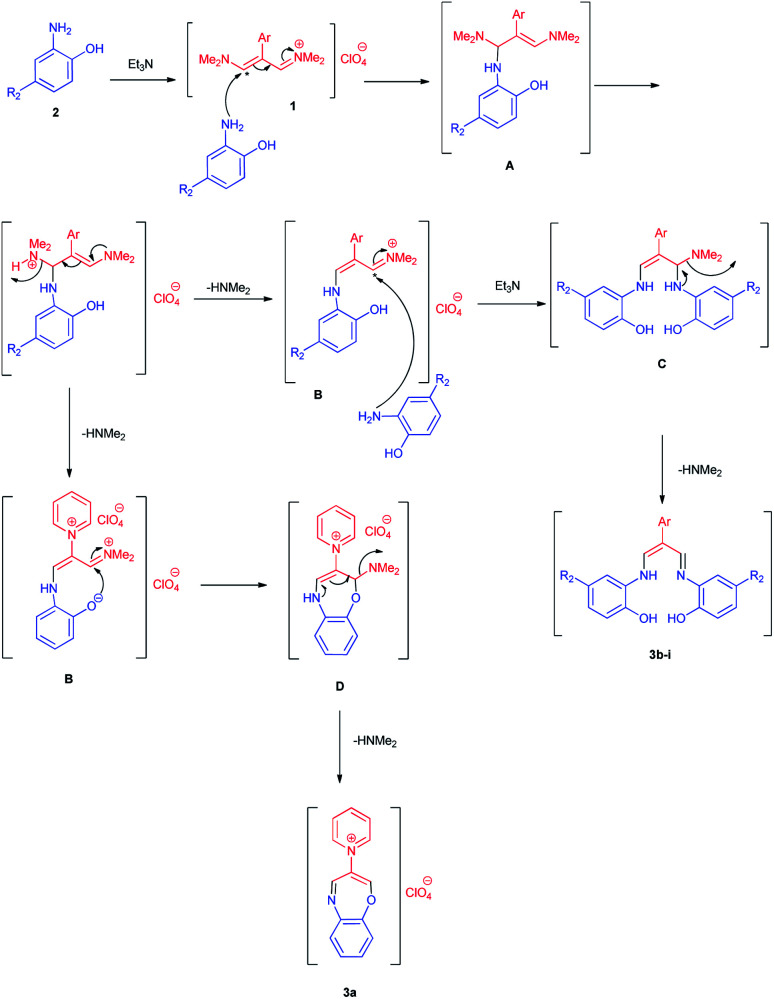
Proposed mechanism for the synthesis product 3a–i.

The proposed mechanism for the formation of Schiff bases 3b–i in the presence of Et_3_N is shown in Scheme 4. First, intermediate A is formed by the nucleophilic attack of the amine group in aminophenol to trimethinium salt 1. Then, removal of dimethylamine occurs, followed by the nucleophilic attack of the second molecule of aminophenol on the obtained iminium salt B to produce intermediate C. The loss of the second dimethylamine molecule in this step yields the desired product. For the formation of 1,4-oxazpine salt 3a, intermediate D is formed by the intramolecular nucleophilic attack of phenolic oxygen on the obtained iminium salt B. The loss of the dimethylamine molecule form intermediate D, yields 3a.

## Experimental

All chemicals were purchased from Merck or Fluka chemical companies. The ^1^H NMR (300 and 400 MHz) and ^13^C NMR spectra (75 and 100 MHz) were run on a Bruker Avance 400. Tetramethylsilane (TMS) was used as the internal standard for the NMR analysis. IR spectra were recorded using an FTIR apparatus. Melting points were recorded on a Stuart Scientific Apparatus SMP3 (UK) in open capillary tubes. Elemental C, H and N analyses, were performed using a Costech CHNS-O elemental analyzer. UV/Vis absorption spectra were recorded at room temperature in DMSO using a Perkin-Elmer Lambda 25 spectrophotometer. MS spectra were recorded with a Agilent 7000 Series Triple Quad-MS mass spectrometer.

### General procedure for the synthesis of Schiff base derivatives 3

A mixture of trimethinium salts 1a–f (1 mmol) and trimethylamine (1.0 mL) were dissolved in boiling ethanol (8 mL). Aminophenols 2a–c (2 mmol) in EtOH (7 mL) were added dropwise to the stirred mixture at reflux ethanol for 12 h. (2-Amino-4-chlorophenol and 2-amino-4-nitrophenol in ethanol (7 mL) and other aminophenols in methanol (7 mL) was dissolved). After completion of the reaction confirmed by TLC, the reaction mixture was set aside in a refrigerator for 12 h. Then the solvent was evaporated under vacuum, and a small amount of dichloromethane (10 mL) was added, and the precipitate formed was collected by filtration, recrystallized from 2-propanol, and dried in vacuum at 80 °C to afford the pure product 3a–i.

### General procedure for the synthesis metal complexes of Schiff base 3c

A solution of Schiff base 3c (1 mmol) and acetate salt of transition metals copper(ii), zinc(ii), cobalt(ii) or nickel(ii) (1 mmol) in DMSO (3 mL) was heated at 100 °C for 6 h. After completion of the reaction confirmed by TLC, the reaction mixture was cooled to room temperature, then a small amount of water (3 mL) was added and the precipitate formed was collected by filtration, recrystallized from distilled water (5 mL) to get pure products in excellent yield.

### 1-(Benzo[*b*][1,4]oxazepin-3-yl)pyridinium perchlorate 3a

Red powder, mp > 260 °C, ^1^H NMR (DMSO-*d*_6_, 400 MHz) *δ* (ppm): 6.79 (d, *J* = 6.4 Hz, 2H), 6.88–6.95 (m, 2H), 7.56 (s, 1H), 8.24 (t, *J* = 7.2 Hz, 2H), 8.27 (s, 1H), 8.72 (t, *J* = 7.8 Hz, 1H), 9.11 (d, *J* = 5.6 Hz, 2H). ^13^C NMR (DMSO-*d*_6_, 100 MHz) *δ* (ppm): 114.3, 115.8, 119.7, 119.9, 125.8, 128.0, 138.8, 146.9, 147.8, 149.2, 150.4, 156.0. Anal. calcd for (C_14_H_11_N_2_O)(ClO_4_): C, 52.11; H, 3.44; N, 8.68%. Found: C, 52.13; H, 3.45; N, 8.67%. *λ*_max_ (DMSO) = 340 nm.

### 1-((2-Hydroxyphenyl)amino)-3-(((2-hydroxyphenyl)imino)propen-2-yl)-3,5-dimethylpyridinium perchlorate 3b

Red powder, mp > 260 °C, ^1^H NMR (DMSO-*d*_6_, 300 MHz) *δ* (ppm): 2.52 (s, 6H), 3.62–4.52 (broad, 3H), 6.72 (t, *J* = 7.2 Hz, 2H), 6.82 (d, *J* = 8.0 Hz, 4H), 6.93 (t, *J* = 7.4 Hz, 2H), 8.28 (s, 2H), 8.63 (s, 1H), 8.99 (s, 2H). ^13^C NMR (DMSO-*d*_6_, 100 MHz) *δ* (ppm): 18.2, 116.8, 117.8, 118.2, 126.1, 133.0, 138.1, 142.2, 145.3, 145.8, 147.5, 152.1. Anal. calcd for (C_22_H_22_N_3_O_2_)(ClO_4_): C, 57.46; H, 4.82; N, 9.14%. Found: C, 57.44; H, 4.83; N, 9.15%. IR (KBr) (*ν*_max_, cm^−1^): 3508, 3365, 1645, 1099 cm^−1^. *λ*_max_ (DMSO) = 355 nm.

### 1-((2-Hydroxy-5-methylphenyl)amino)-3-(((2-hydroxy-5-methylphenyl)imino)propen-2-yl)-3,5-dimethylpyridinium perchlorate 3c

Orang powder, mp > 260 °C, ^1^H NMR (DMSO-*d*_6_, 300 MHz) *δ* (ppm): 2.27 (s, 6H), 2.54 (s, 6H), 6.78–6.85 (m, 4H), 7.27 (s, 2H), 8.37 (d, 1H), 8.60 (s, 2H), 9.01 (s, 2H), 9.39 (broad, 2H), 12.34 (s, 1H). ^13^C NMR (DMSO-*d*_6_, 75 MHz) *δ* (ppm): 18.2, 20.9, 116.0, 118.2, 118.8, 126.5, 129.1, 132.4, 138.2, 142.6, 145.8, 146.5, 147.0. Anal. calcd for (C_24_H_26_N_3_O_2_)(ClO_4_): C, 59.08; H, 5.37; N, 8.61%. Found: C, 59.10; H, 5.39; N, 8.60%. IR (KBr) (*ν*_max_, cm^−1^): 3500, 1645, 1375, 1090 cm^−1^. MS *m*/*z* calcd for C_24_H_26_N_3_O_2_ [M]^+^ 388.4, found 388.2. *λ*_max_ (DMSO) = 370 nm.

### 4-Chloro-3-((5-chloro-2-hydroxyphenyl)amino)-2-(((naphthalen-1-yl)allylidene)amino)phenol 3d

Yellow powder, mp > 260 °C, ^1^H NMR (DMSO-*d*_6_, 300 MHz) *δ* (ppm): 6.87 (d, *J* = 8.4 Hz, 2H), 7.09 (dd, *J* = 1.6, 8.5 Hz, 1H), 7.43 (s, 1H), 7.59–7.73 (m, 5H), 7.84 (m, 2H), 8.09–8.15 (m, 2H), 8.35 (s, 1H), 8.74 (s, 1H), 10.36 (broad, 1H). ^13^C NMR (DMSO-*d*_6_, 75 MHz) *δ* (ppm): 106.9, 117.8, 119.3, 123.5, 125.0, 126.4, 126.6, 127.3, 128.0, 128.1, 128.3, 129.3, 130.3, 130.4, 132.3, 134.0, 147.1, 159.2, 164.7. Anal. calcd for C_25_H_18_Cl_2_N_2_O_2_: C, 66.83; H, 4.04; N, 6.23%. Found: C, 66.85; H, 4.05; N, 6.21%. MS *m*/*z* calcd for C_25_H_18_Cl_2_N_2_O_2_ [M]^+^ 449.3, found 449. *λ*_max_ (DMSO) = 340 nm.

### 3-((2-Hydroxy-5-methylphenyl)amino)-2-((phenylallylidene)amino)-4-methylphenol 3e

Yellow powder, mp > 260 °C, ^1^H NMR (DMSO-*d*_6_, 300 MHz) *δ* (ppm): 2.27 (s, 6H), 6.88–6.95 (m, 4H), 7.27 (s, 2H), 7.49–7.68 (m, 5H), 8.79 (s, 1H), 9.61–9.62 (m, 1H), 10.21 (s, 1H). ^13^C NMR (DMSO-*d*_6_, 75 MHz) *δ* (ppm): 20.7, 111.8, 116.6, 120.4, 126.3, 128.4, 129.0, 129.2, 129.9, 130.6, 130.7, 140.5, 158.6. Anal. calcd for C_23_H_22_N_2_O_2_: C, 77.07; H, 6.19; N, 7.82%. Found: C, 77.06; H, 6.21; N, 7.80%. *λ*_max_ (DMSO) = 335 nm.

### 4-Chloro-3-((5-chloro-2-hydroxyphenyl)amino)-2-((phenylallylidene)amino)phenol 3f

Brown powder, mp > 260 °C, ^1^H NMR (DMSO-*d*_6_, 400 MHz) *δ* (ppm): 6.86 (t, *J* = 7.4 Hz, 2H), 6.92–6.99 (m, 3H), 7.42 (d, *J* = 7.6 Hz, 2H), 7.46 (d, *J* = 7.6 Hz, 2H), 7.53 (s, 2H), 8.39 (d, *J* = 6 Hz, 2H), 9.39 (broad, 2H), 12.85 (s, 1H). ^13^C NMR (DMSO-*d*_6_, 100 MHz) *δ* (ppm): 111.7, 115.0, 122.5, 127.5, 127.7, 128.0, 129.1, 129.5, 129.7, 130.5, 140.6, 152.6. Anal. calcd for C_21_H_16_Cl_2_N_2_O_2_: C, 63.17; H, 4.04; N, 7.02%. Found: C, 63.15; H, 4.05; N, 7.01%. *λ*_max_ (DMSO) = 330 nm.

### 2-(4-Bromophenyl)-3-((((5-chloro-2-hydroxyphenyl)amino)allylidene)amino)-4-chlorophenol 3g

Brown powder, mp > 260 °C, ^1^H NMR (DMSO-*d*_6_, 300 MHz) *δ* (ppm): 6.90 (d, *J* = 8.4 Hz, 2H), 6.97 (dd, *J* = 2.2, 8.5 Hz, 2H), 7.52–7.59 (m, 6H), 8.41 (d, *J* = 5.7 Hz, 2H), 9.82 (broad, 2H), 12.82 (t, *J* = 5.8 Hz, 1H). ^13^C NMR (DMSO-*d*_6_, 75 MHz) *δ* (ppm): 108.9, 117.1, 117.4, 118.6, 124.0, 124.1, 128.4, 131.7, 135.2, 139.3, 147.2, 149.8. Anal. calcd for C_21_H_15_BrCl_2_N_2_O_2_: C, 52.75; H, 3.16; N, 5.86%. Found: C, 52.78; H, 3.15; N, 5.84%. *λ*_max_ (DMSO) = 360 nm.

### 2-(4-Chlorophenyl)-3-((((5-chloro-2-hydroxyphenyl)amino)allylidene)amino)-4-chlorophenol 3h

Brown powder, mp > 260 °C, ^1^H NMR (DMSO-*d*_6_, 400 MHz) *δ* (ppm): 6.91 (d, *J* = 8.4 Hz, 2H), 6.97 (d, *J* = 8.4 Hz, 2H), 7.38 (s, 2H), 7.58 (d, *J* = 12.4 Hz, 2H), 7.70 (d, *J* = 12 Hz, 2H), 8.45 (d, *J* = 4.8 Hz, 2H), 9.89 (broad, 2H), 12.82 (s, 1H). ^13^C NMR (DMSO-*d*_6_, 100 MHz) *δ* (ppm): 110.0, 117.1, 117.4, 123.8, 123.9, 126.6, 132.7, 133.8, 135.3, 137.0, 147.4, 149.6. Anal. calcd for C_21_H_15_Cl_3_N_2_O_2_: C, 58.15; H, 3.49; N, 6.46%. Found: C, 58.14; H, 3.47; N, 6.47%. *λ*_max_ (DMSO) = 345 nm.

### 2-(4-Chlorophenyl)-3-((((2-hydroxyphenyl)amino)allylidene)amino)phenol 3i

Brown powder, mp > 260 °C, ^1^H NMR (DMSO-*d*_6_, 400 MHz) *δ* (ppm): 6.78–6.89 (m, 8H), 7.00 (d, *J* = 7.2 Hz, 2H), 7.11 (d, *J* = 7.6 Hz, 2H), 8.31 (d, *J* = 5.6 Hz, 2H), 8.99 (broad, 2H), 12.00 (s, 1H). ^13^C NMR (DMSO-*d*_6_, 100 MHz) *δ* (ppm): 115.3, 120.8, 124.7, 126.0, 127.5, 131.3, 133.9, 140.0, 141.3, 150.3, 158.4, 162.2. Anal. calcd for C_21_H_17_ClN_2_O_2_: C, 69.14; H, 4.70; N, 7.68%. Found: C, 69.15; H, 4.72; N, 7.67%. *λ*_max_ (DMSO) = 350 nm.

## Conclusions

In conclusion, we have reported a highly efficient method for the synthesis of important Schiff base derivatives *via* a condensation reaction between 2-substituted trimethinium salts as starting compounds with aminophenol in presence of triethylamine in ethanol at reflux. A simple procedure in the excellent yields, mild reaction conditions, and metal-catalyst free are the main advantages of this method. Metal complexes of Schiff base can be used as catalysts and to advance a number of reactions such as carbon–carbon or carbon–nitrogen coupling reactions, which are separately planned for future works.

## Conflicts of interest

There are no conflicts to declare.

## Supplementary Material

RA-011-D1RA04214A-s001
