# Supplementary material for: Synthesis of new allylidene amino phenol-containing Schiff bases and metal complex formation using trimethinium salts
Source: RSC Adv. 2021 Jun 18;11(35):21695–701. doi: 10.1039/d1ra04214a (PMC9034104; doi:10.1039/d1ra04214a)
Supplement: RA-011-D1RA04214A-s001 [file RA-011-D1RA04214A-s001.pdf]

Electronic Supplementary Material (ESI) for RSC Advances.
This journal is © The Royal Society of Chemistry 2021

Synthesis of new allylidene amino phenol-containing Schiff bases and metal complexes formation using trimethinium salts

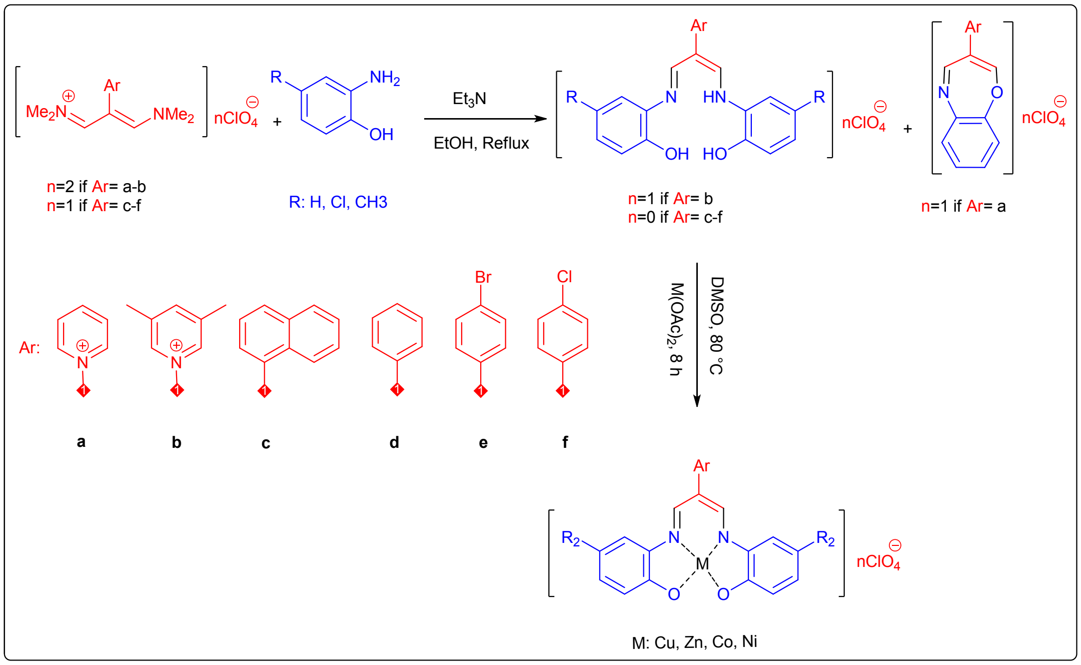

1-(Benzo[b][1,4]oxazepin-3-yl)pyridinium perchlorate 3a:

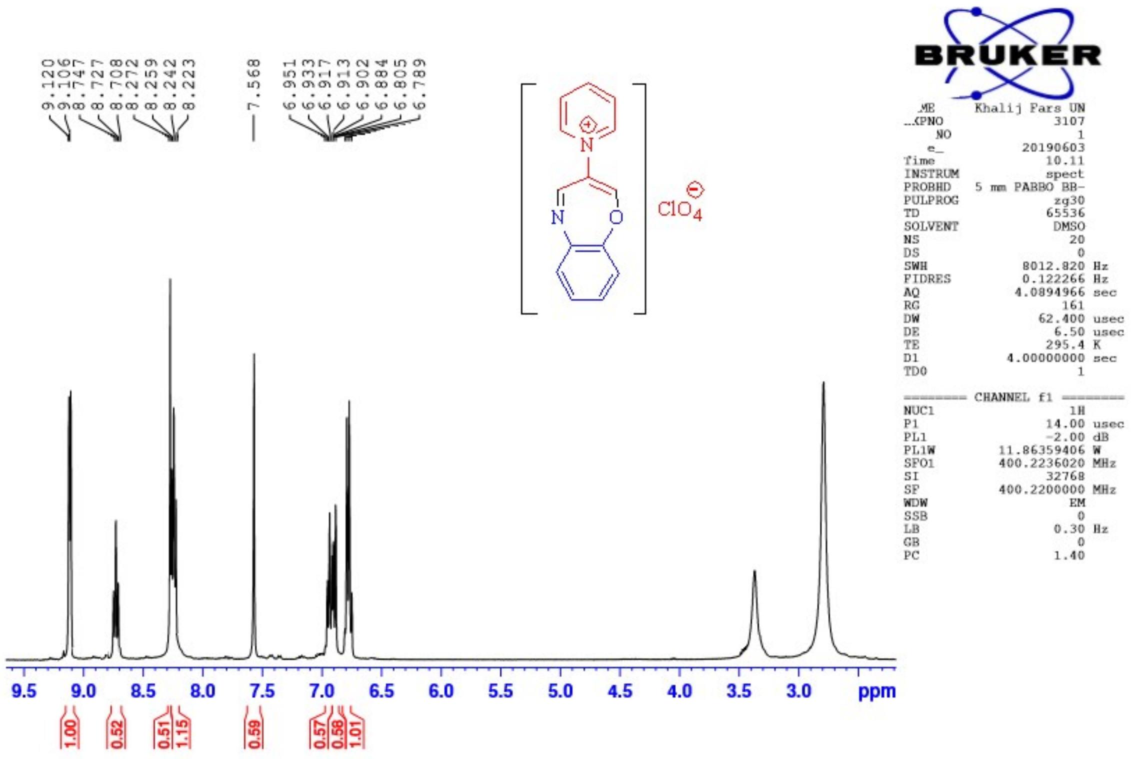

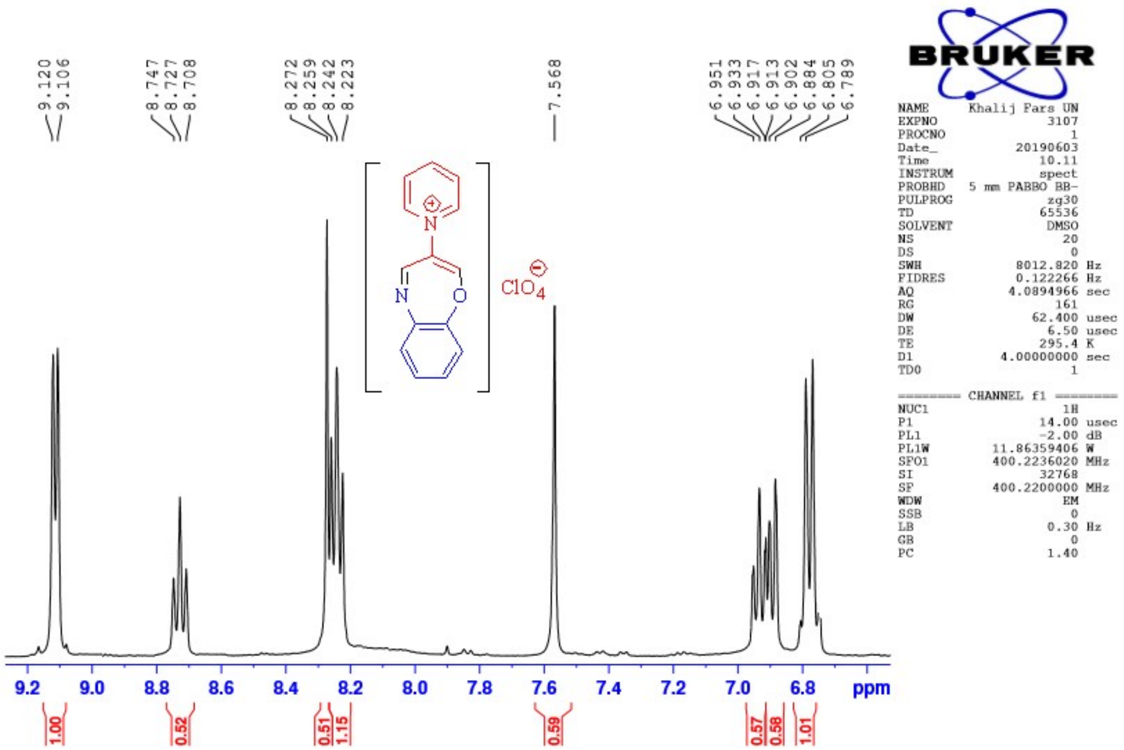

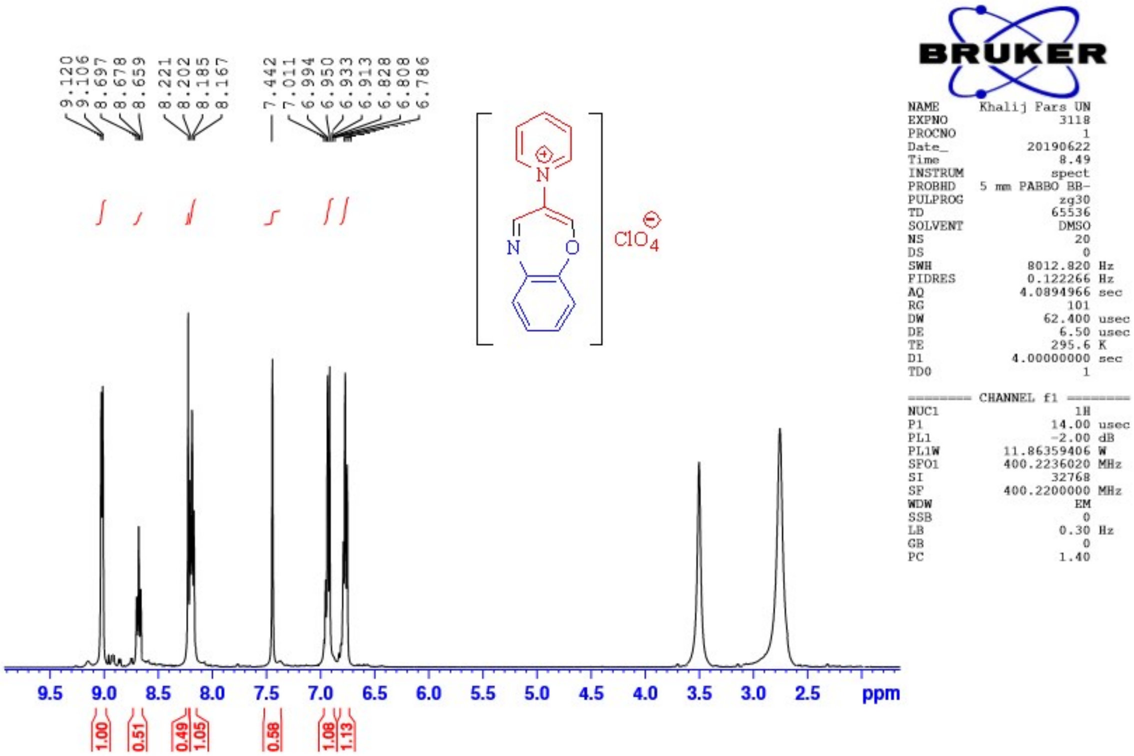

(Exchange in D_2O)

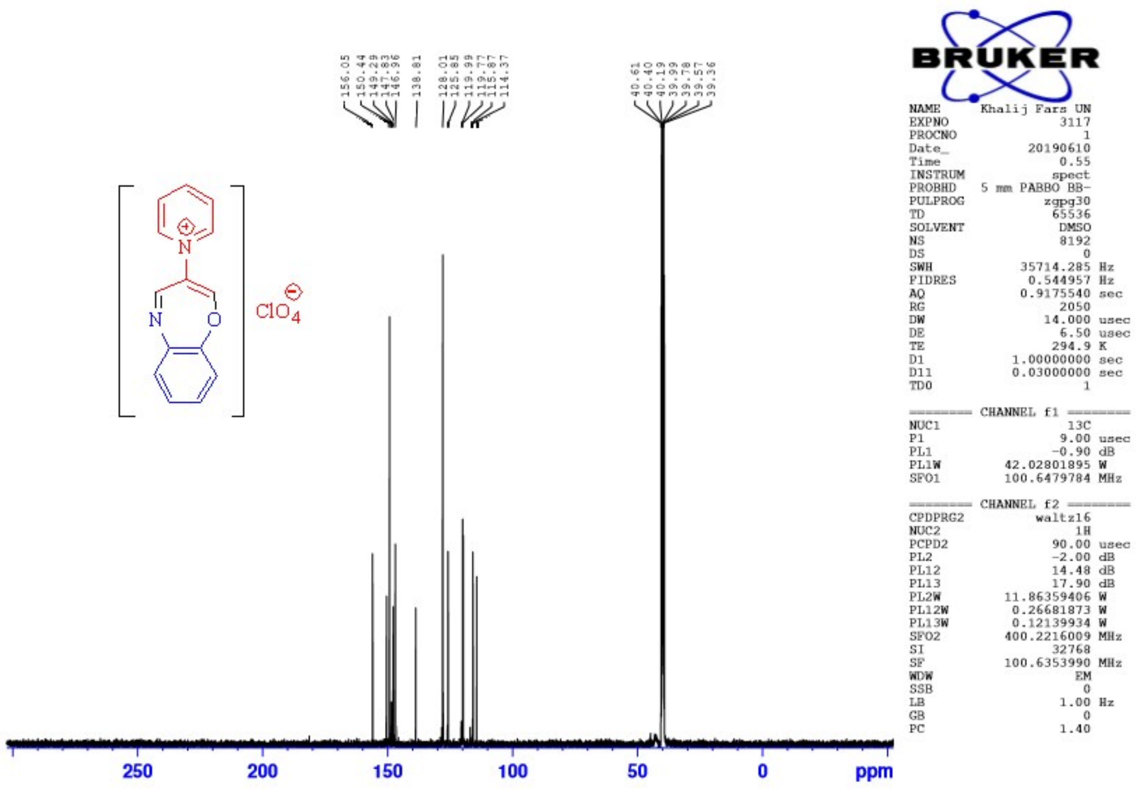

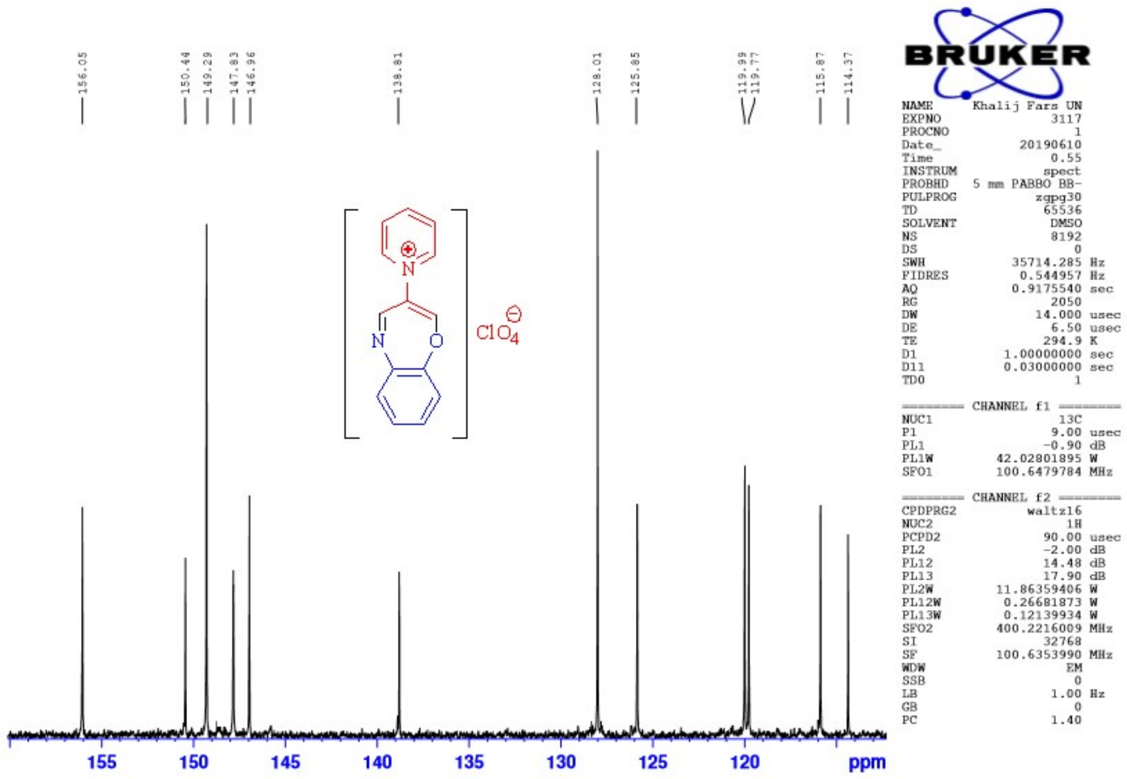

1-((2-Hydroxyphenyl)amino)-3-((2-hydroxyphenyl)imino)propen-2-yl)-3,5-dimethylpyridinium perchlorate 3b:

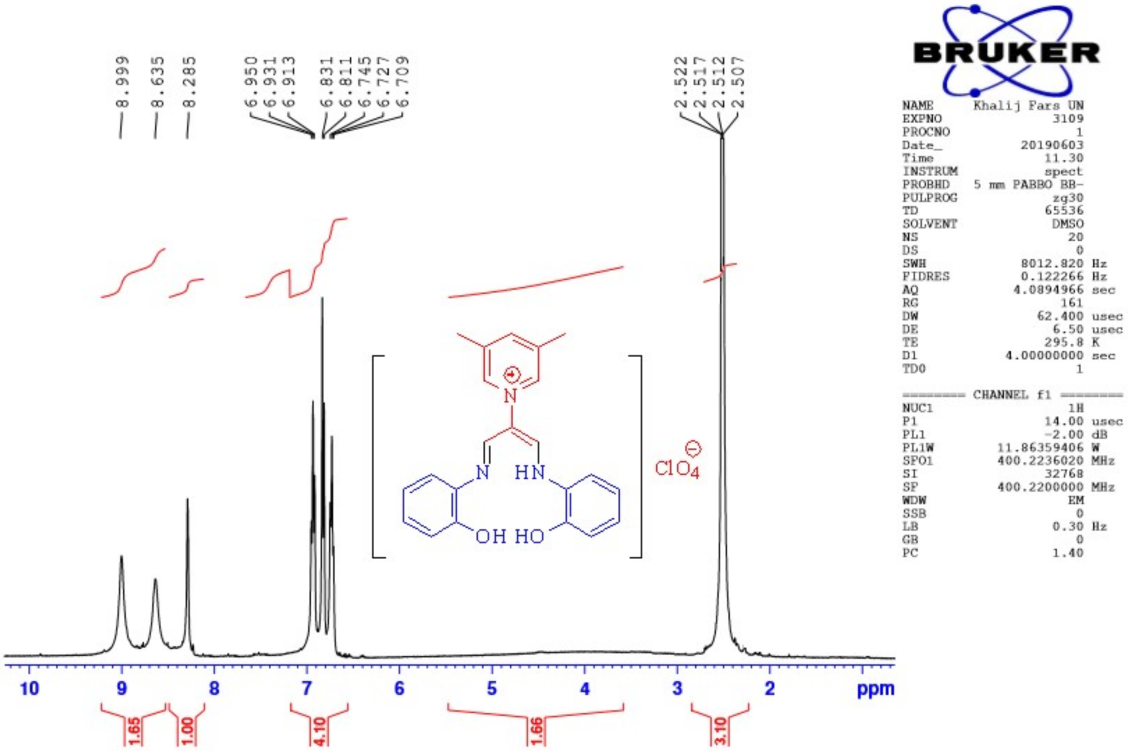
8.999
8.635
8.285
6.950
6.931
6.913
6.831
6.811
6.745
6.727
6.709
2.522
2.517
2.512
2.507
N
HN
OH HO
ClO4
10 9 8 7 6 5 4 3 2 ppm
1.65
1.00
4.10
1.66
3.10
BRUKER
NAME Khalij Fars UN
EXPNO 3109
PROCNO 1
Date_ 20190603
Time 11.30
INSTRUM spect
PROBHD 5 mm PABBO BB-
PULPROG zg30
TD 65536
SOLVENT DMSO
NS 20
DS 0
SWH 8012.820 Hz
FIDRES 0.122266 Hz
AQ 4.0894966 sec
RG 161
DW 62.400 usec
DE 6.50 usec
TE 295.8 K
D1 4.00000000 sec
TD0 1
======== CHANNEL f1 ========
NUC1 1H
P1 14.00 usec
PL1 -2.00 dB
PL1W 11.86359406 W
SFO1 400.2236020 MHz
SI 32768
SF 400.2200000 MHz
WDW EM
SSB 0
LB 0.30 Hz
GB 0
PC 1.40

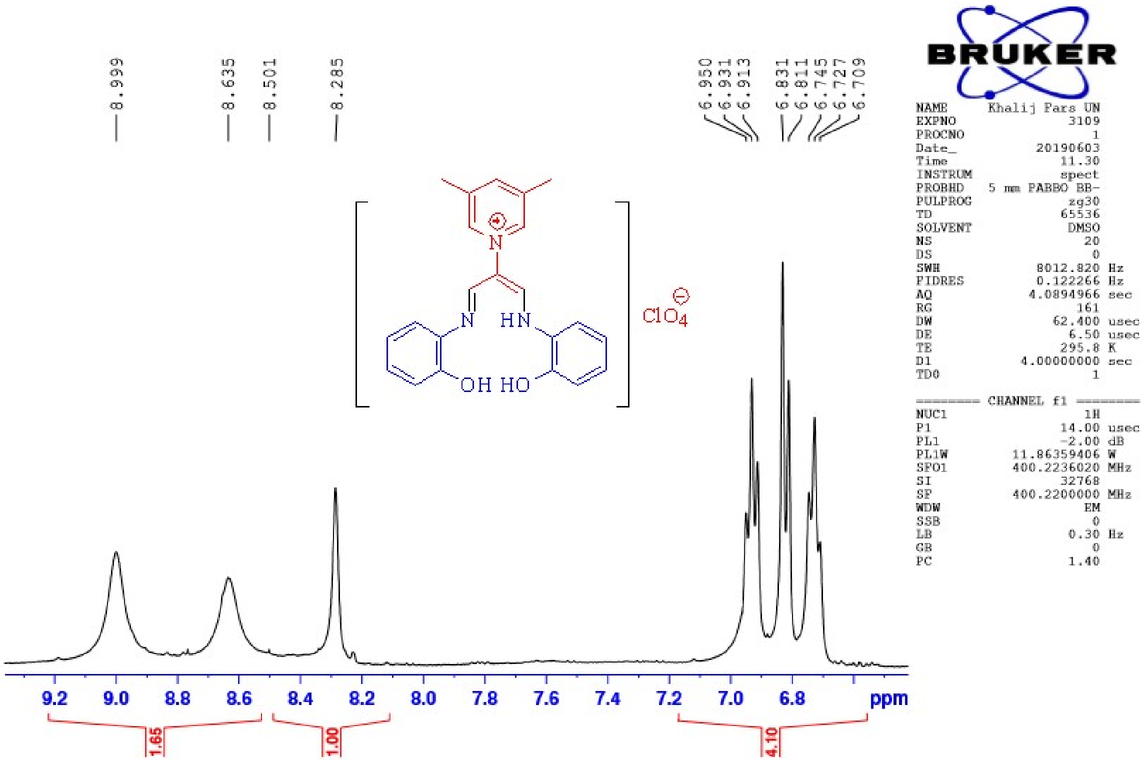
8.999
8.635
8.501
8.285
6.950
6.931
6.913
6.831
6.811
6.745
6.727
6.709
N
HN
OH HO
ClO4
9.2 9.0 8.8 8.6 8.4 8.2 8.0 7.8 7.6 7.4 7.2 7.0 6.8 ppm
1.65
1.00
4.10
BRUKER
NAME Khalij Fars UN
EXPNO 3109
PROCNO 1
Date_ 20190603
Time 11.30
INSTRUM spect
PROBHD 5 mm PABBO BB-
PULPROG zg30
TD 65536
SOLVENT DMSO
NS 20
DS 0
SWH 8012.820 Hz
FIDRES 0.122266 Hz
AQ 4.0894966 sec
RG 161
DW 62.400 usec
DE 6.50 usec
TE 295.8 K
D1 4.00000000 sec
TD0 1
======== CHANNEL f1 ========
NUC1 1H
P1 14.00 usec
PL1 -2.00 dB
PL1W 11.86359406 W
SFO1 400.2236020 MHz
SI 32768
SF 400.2200000 MHz
WDW EM
SSB 0
LB 0.30 Hz
GB 0
PC 1.40

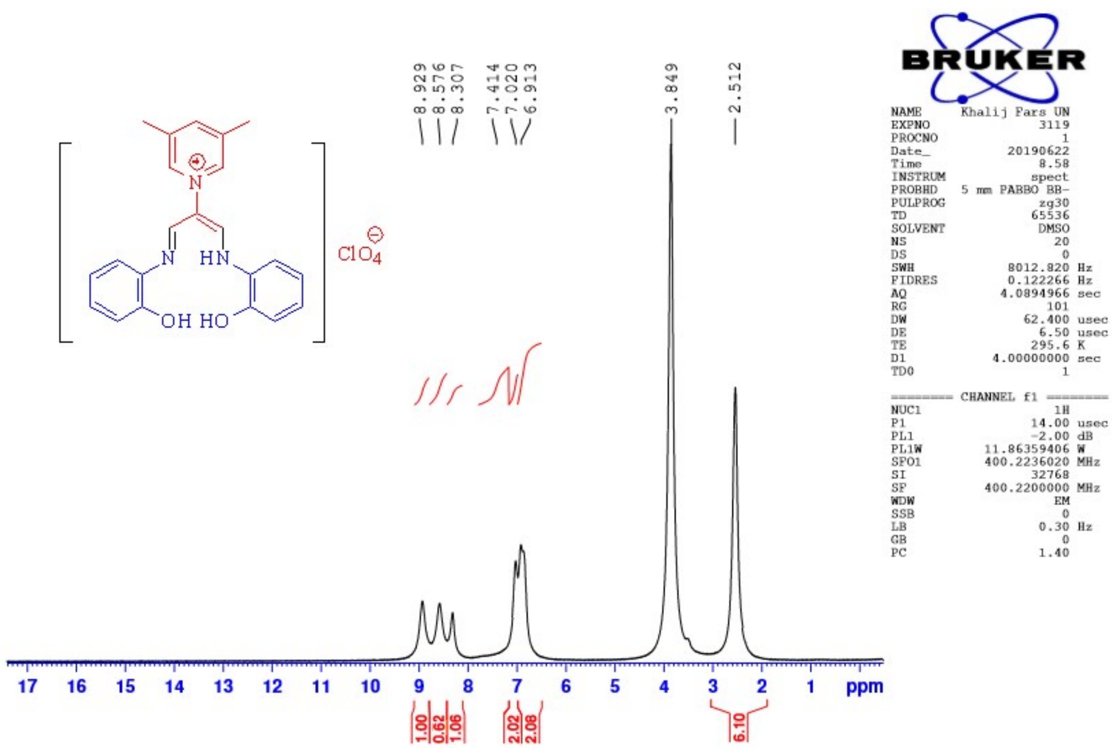

(Exchange in D_2O)

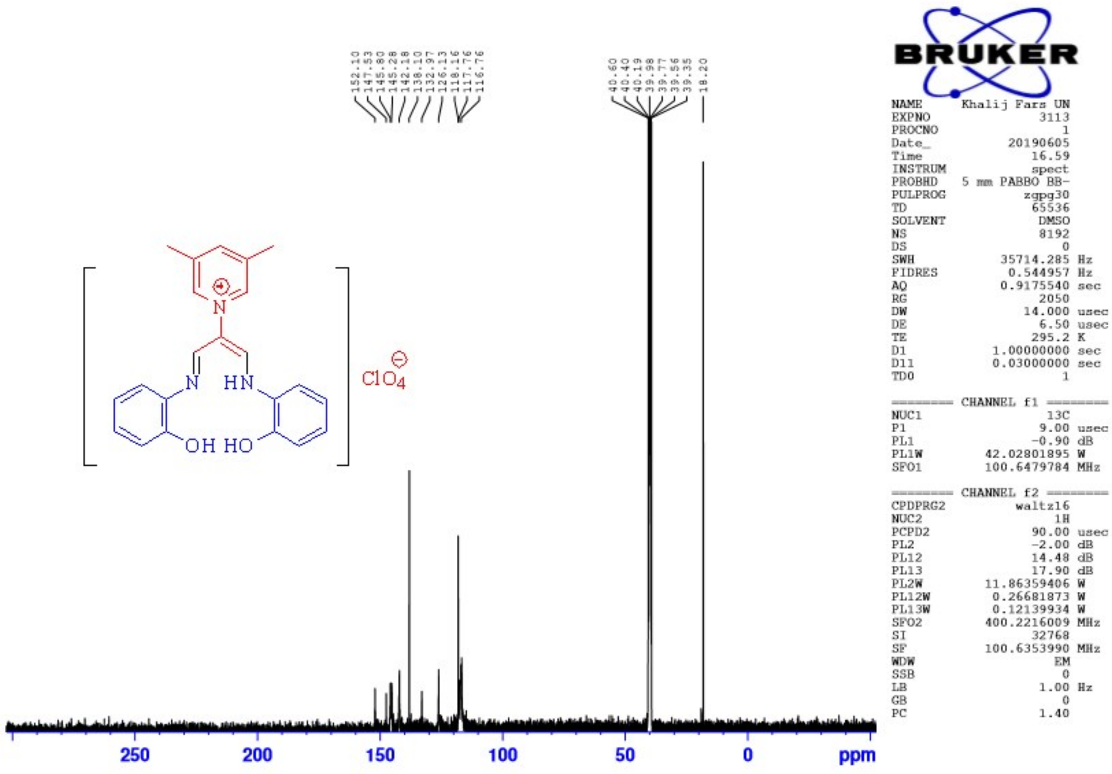

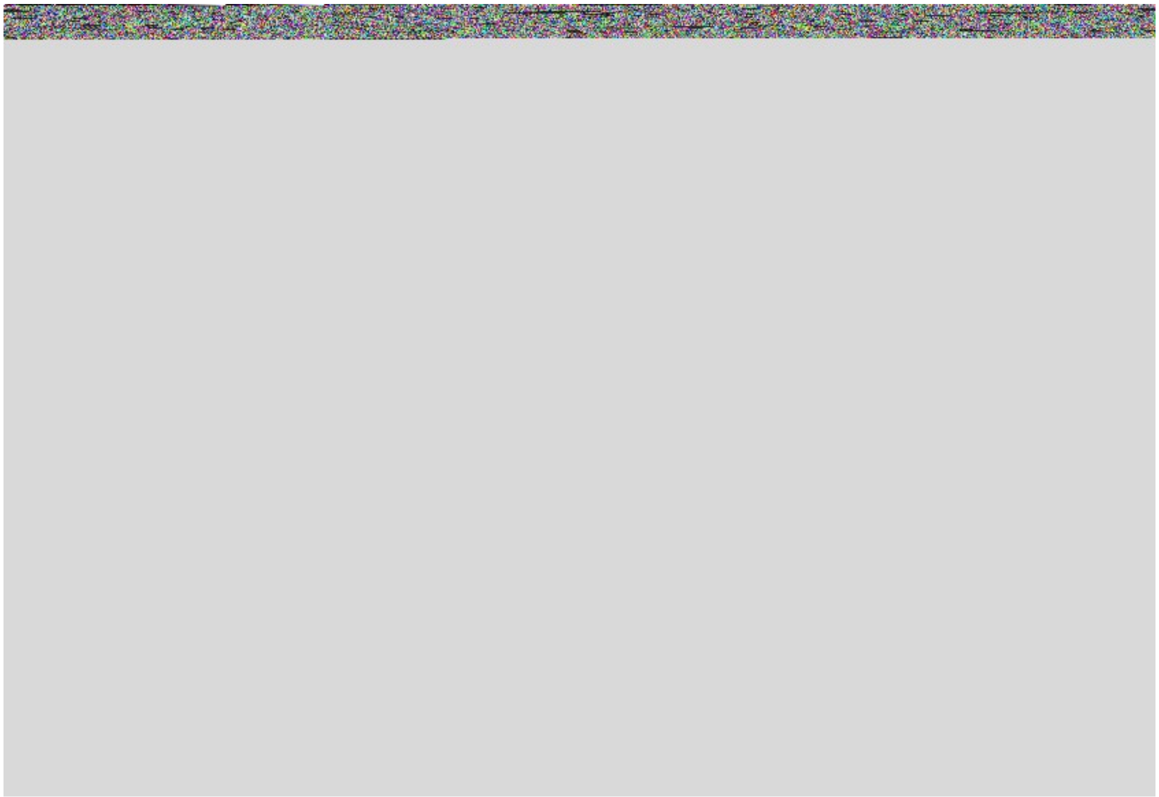

1-((2-Hydroxy-5-methylphenyl)amino)-3-((2-hydroxy-5-methylphenyl)imino) propen-2-yl)-3,5-dimethylpyridinium perchlorate 3c:

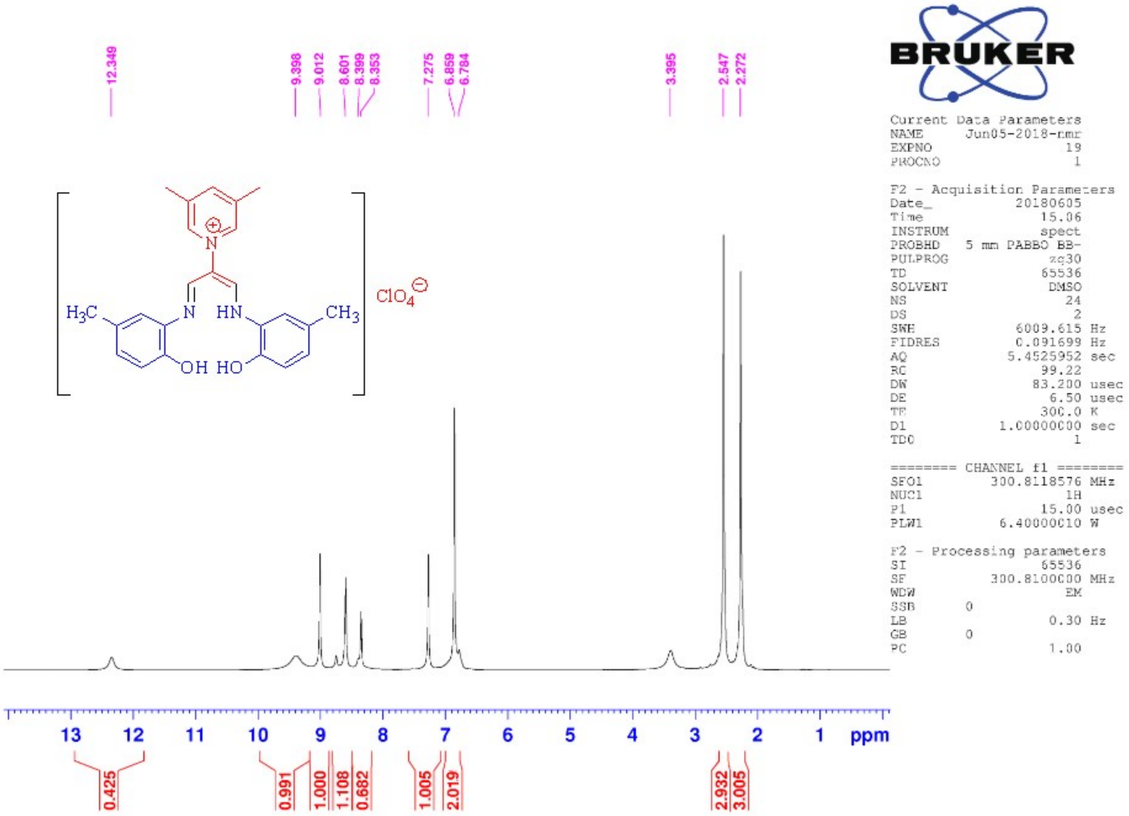

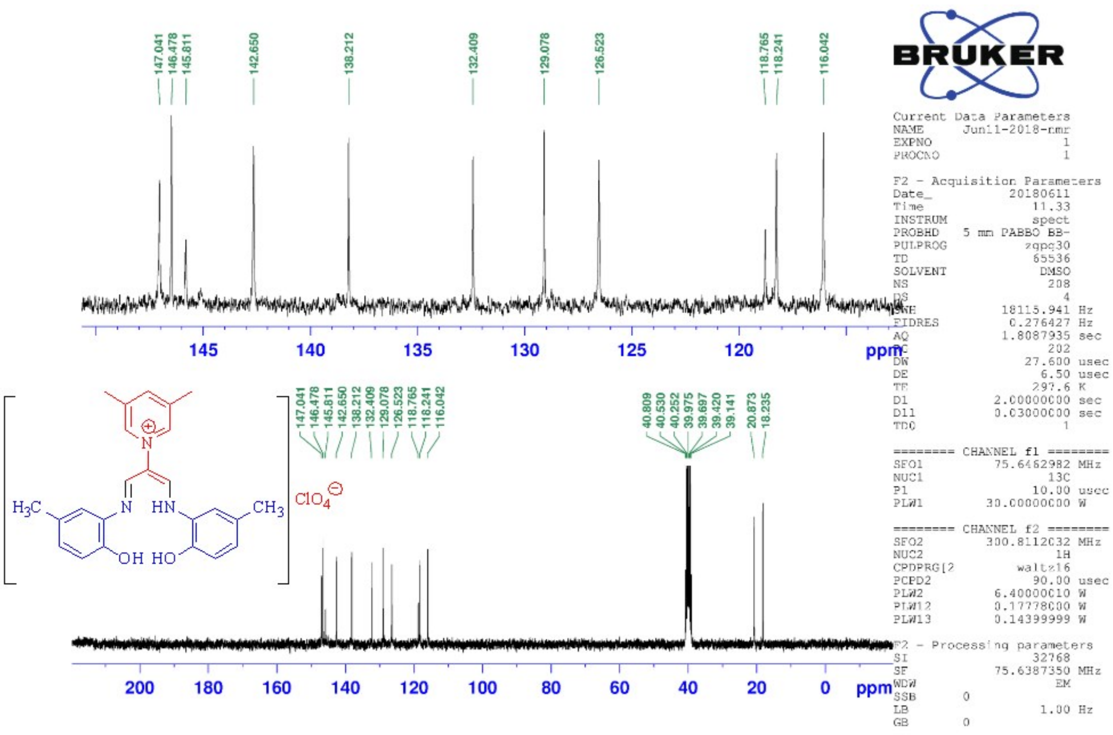

4-Chloro-3-((5-chloro-2-hydroxyphenyl)amino)-2-(naphthalen-1-yl)allylidene) amino)phenol 3d:

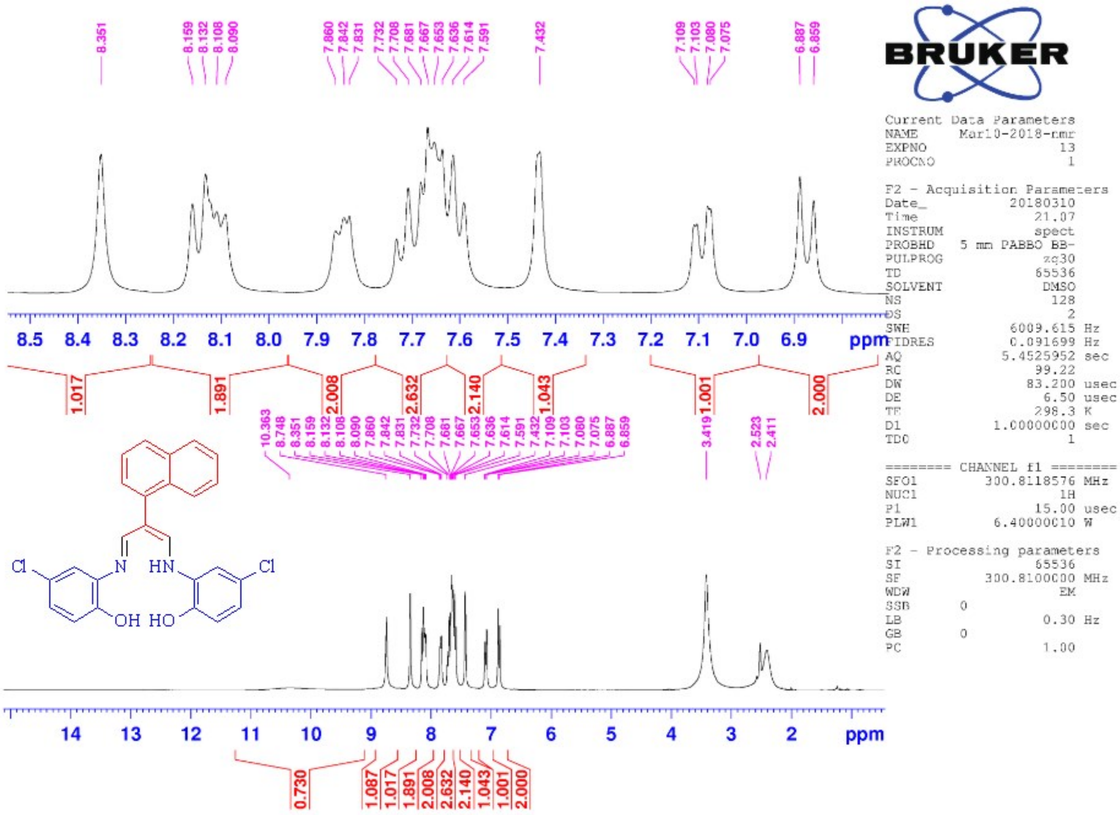

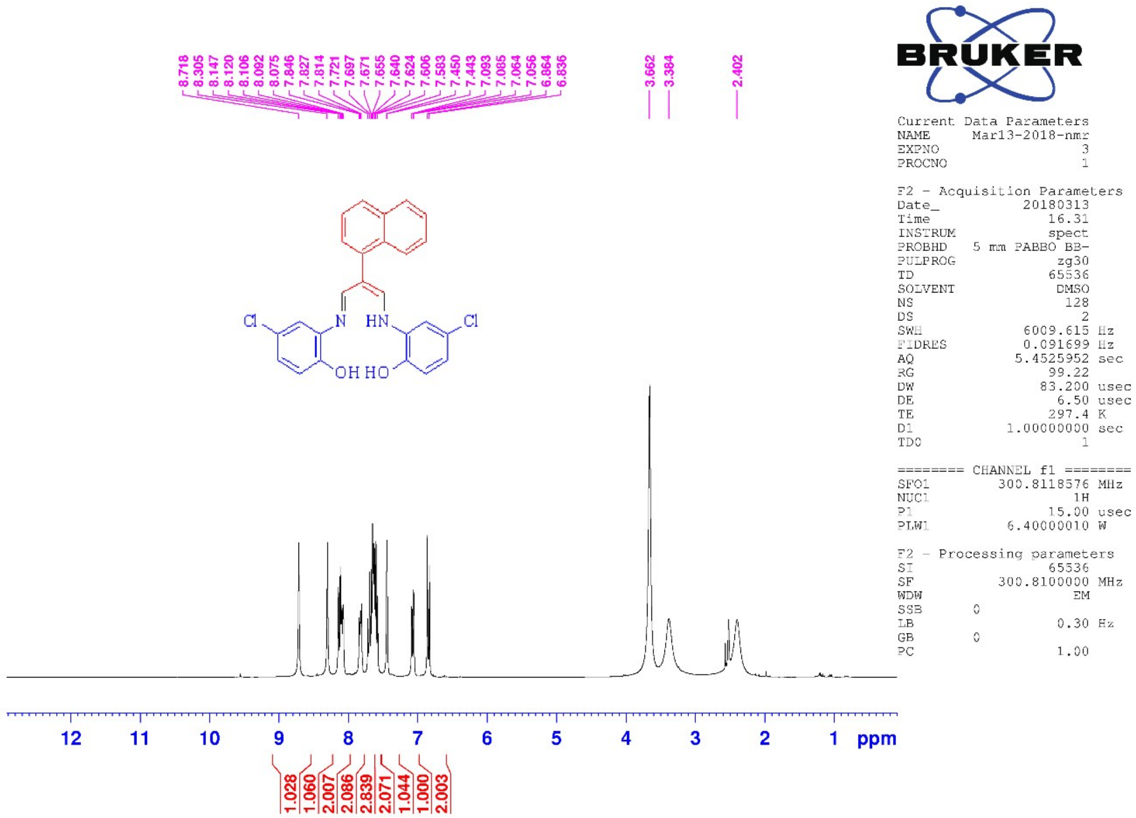

(Exchange in D_2O)

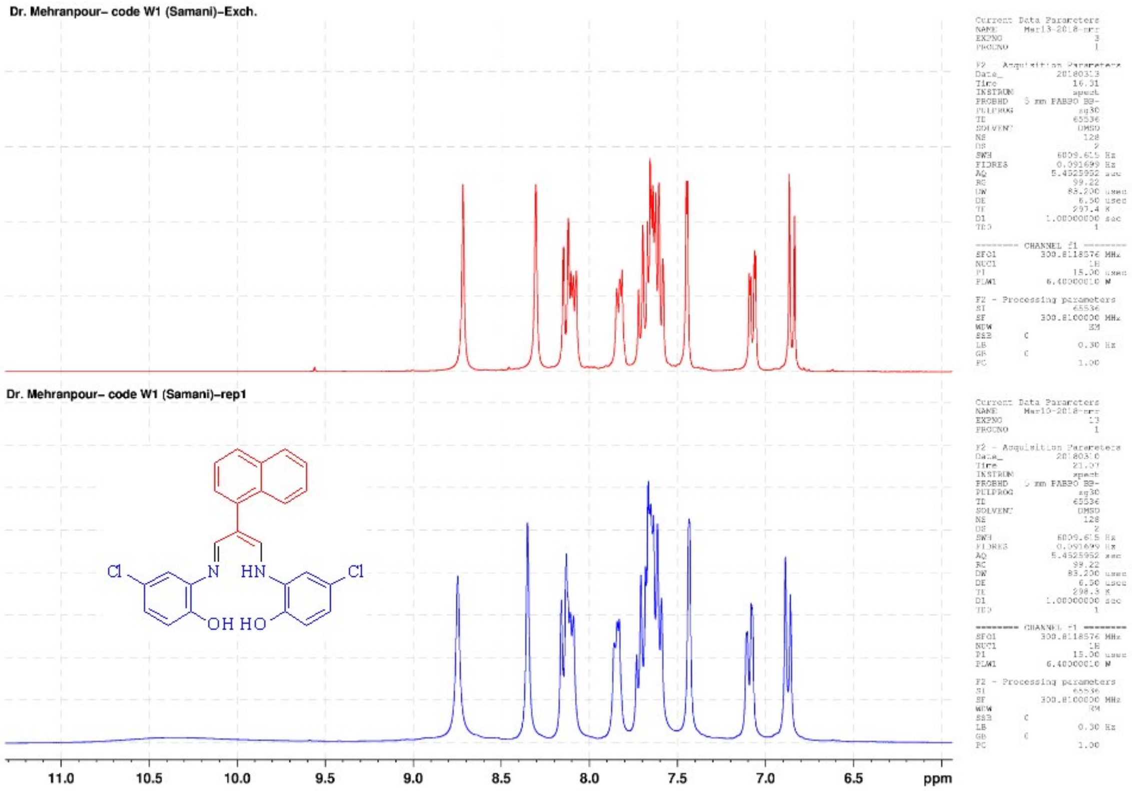

(Exchange in D_2O)

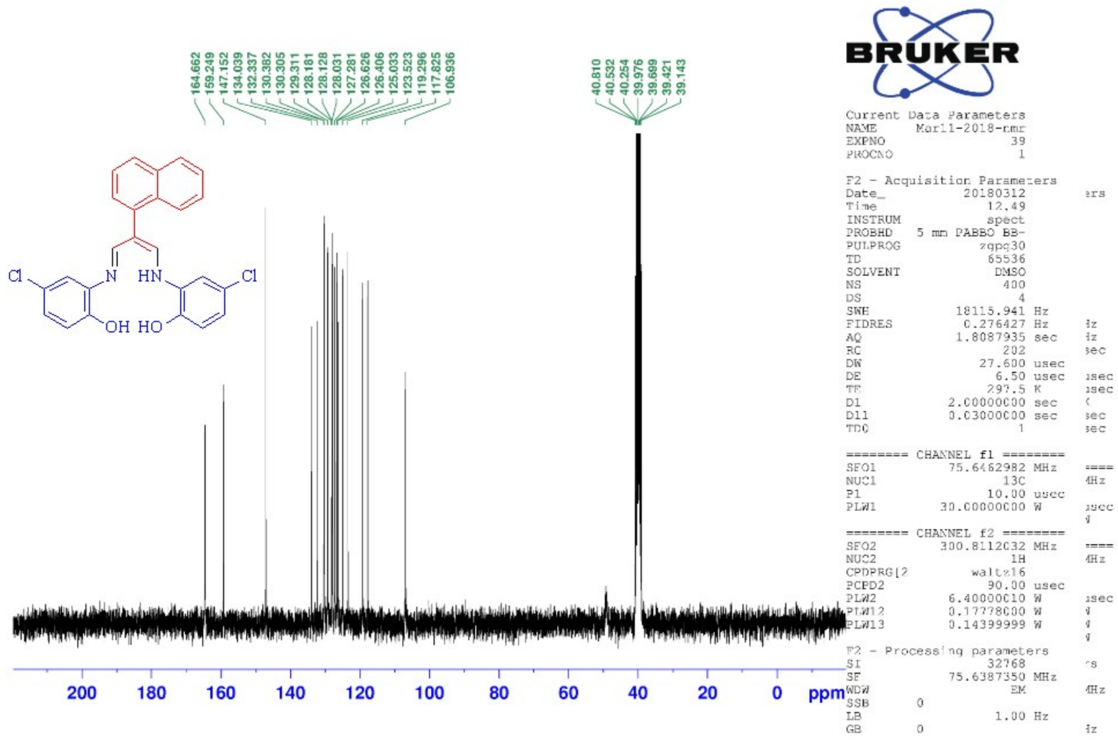

3-((2-Hydroxy-5-methylphenyl)amino)-2-phenylallylidene)amino)-4-methylphenol 3e:

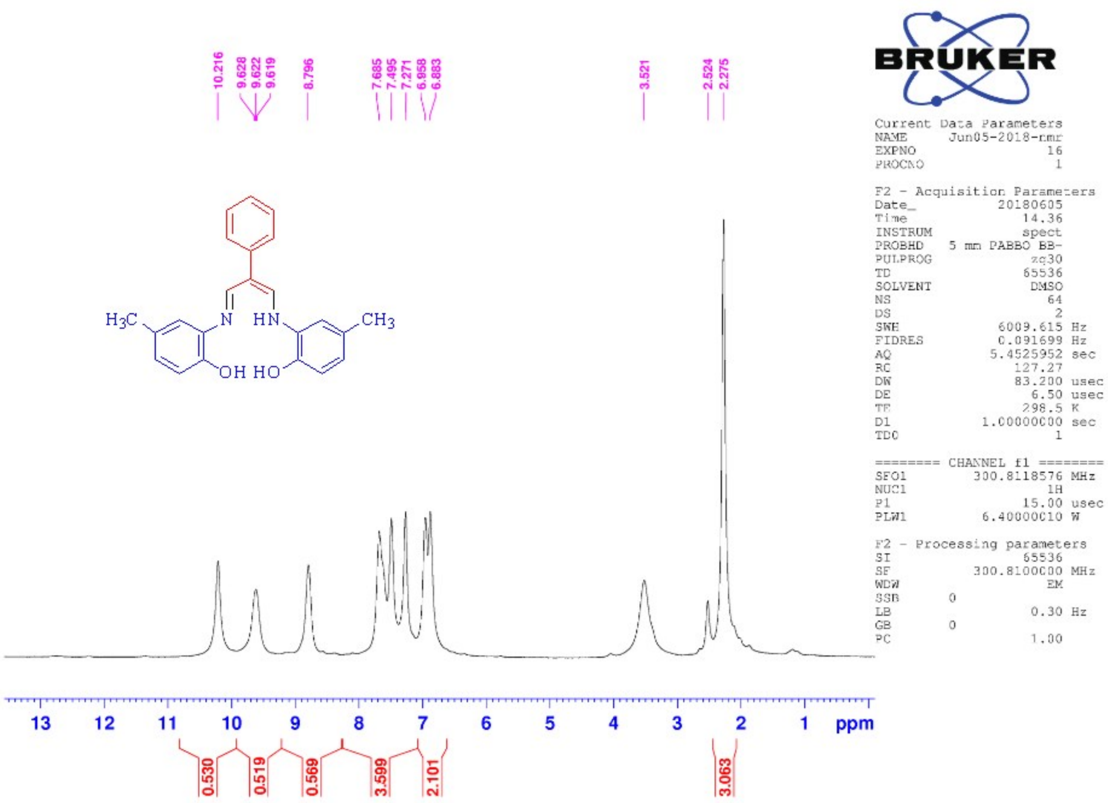

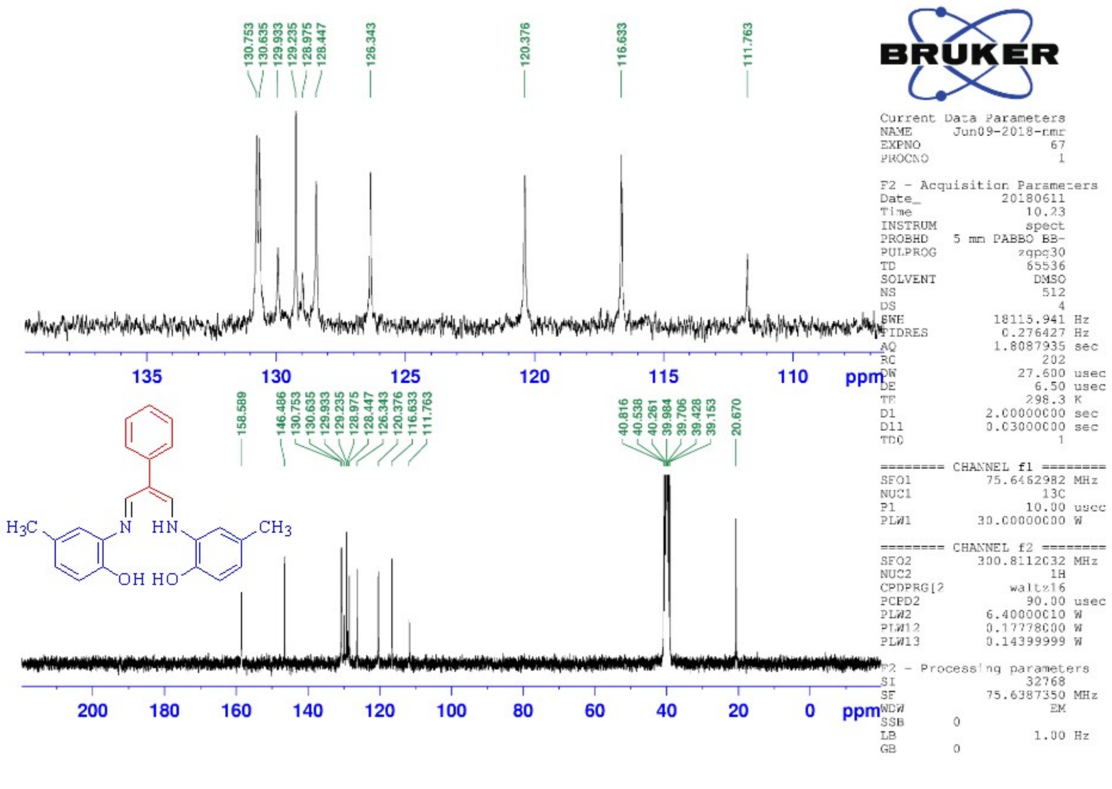

4-Chloro-3-((5-chloro-2-hydroxyphenyl)amino)-2-phenylallylidene) amino) phenol 3f:

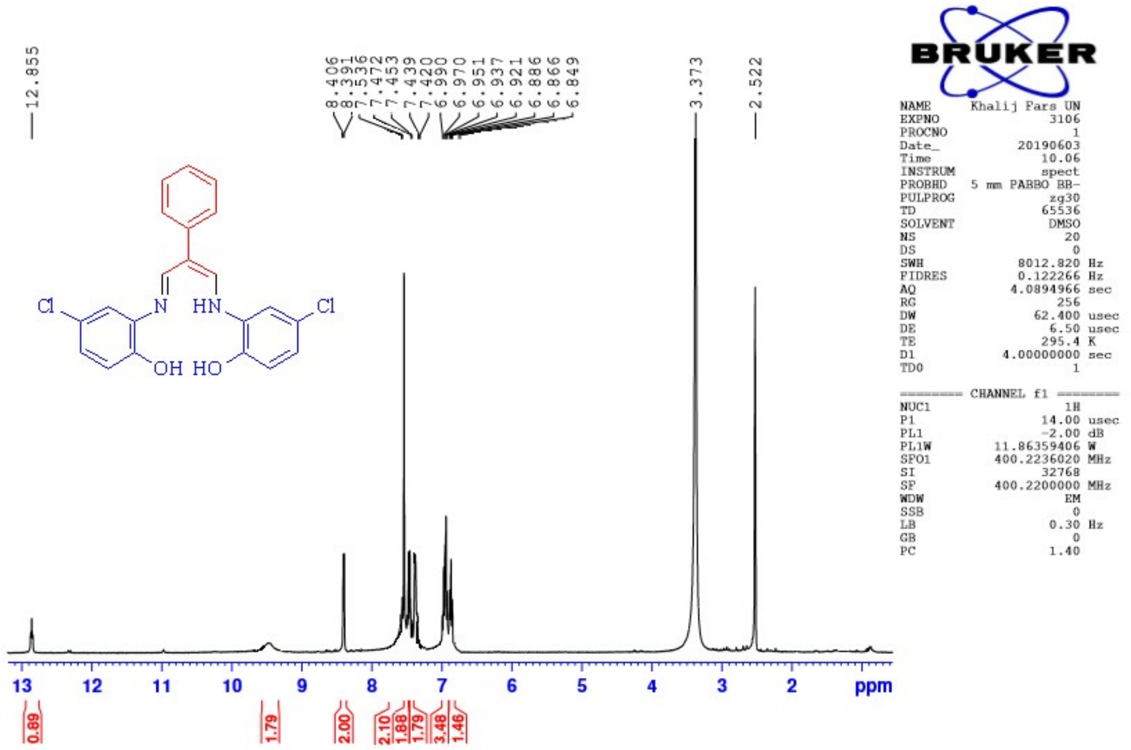
12.855
8.406
8.391
7.536
7.472
7.453
7.439
7.420
6.990
6.970
6.951
6.937
6.921
6.886
6.866
6.849
3.373
2.522
Cl
N
HN
Cl
OH HO
13 12 11 10 9 8 7 6 5 4 3 2 ppm
0.89
1.79
2.00
2.10
1.88
1.79
3.48
1.46
BRUKER
NAME Khalij Fars UN
EXPNO 3106
PROCNO 1
Date_ 20190603
Time 10.06
INSTRUM spect
PROBHD 5 mm PABBO BB-
PULPROG zg30
TD 65536
SOLVENT DMSO
NS 20
DS 0
SWH 8012.820 Hz
FIDRES 0.122266 Hz
AQ 4.0894966 sec
RG 256
DW 62.400 usec
DE 6.50 usec
TE 295.4 K
D1 4.00000000 sec
TD0 1
======== CHANNEL f1 ========
NUC1 1H
P1 14.00 usec
PL1 -2.00 dB
PL1W 11.86359406 W
SFO1 400.2236020 MHz
SI 32768
SF 400.2200000 MHz
WDW EM
SSB 0
LB 0.30 Hz
GB 0
PC 1.40

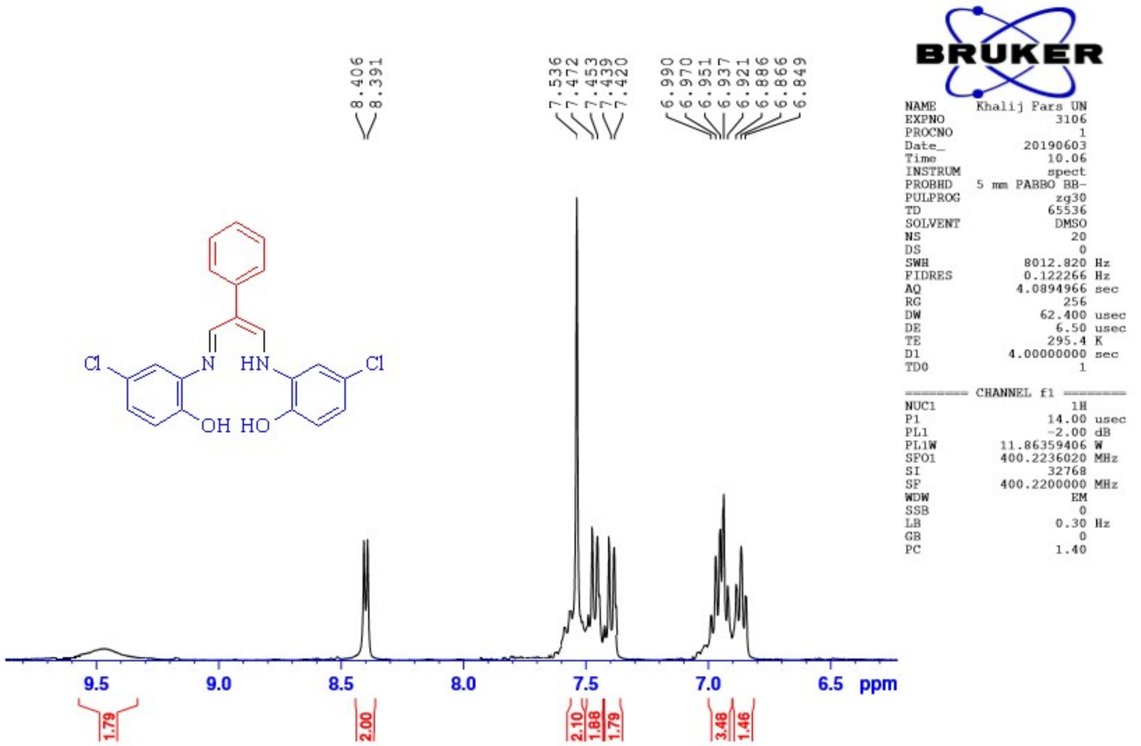
8.406
8.391
7.536
7.472
7.453
7.439
7.420
6.990
6.970
6.951
6.937
6.921
6.886
6.866
6.849
Cl
N
HN
Cl
OH HO
9.5 9.0 8.5 8.0 7.5 7.0 6.5 ppm
1.79
2.00
2.10
1.88
1.79
3.48
1.46
BRUKER
NAME Khalij Fars UN
EXPNO 3106
PROCNO 1
Date_ 20190603
Time 10.06
INSTRUM spect
PROBHD 5 mm PABBO BB-
PULPROG zg30
TD 65536
SOLVENT DMSO
NS 20
DS 0
SWH 8012.820 Hz
FIDRES 0.122266 Hz
AQ 4.0894966 sec
RG 256
DW 62.400 usec
DE 6.50 usec
TE 295.4 K
D1 4.00000000 sec
TD0 1
======== CHANNEL f1 ========
NUC1 1H
P1 14.00 usec
PL1 -2.00 dB
PL1W 11.86359406 W
SFO1 400.2236020 MHz
SI 32768
SF 400.2200000 MHz
WDW EM
SSB 0
LB 0.30 Hz
GB 0
PC 1.40

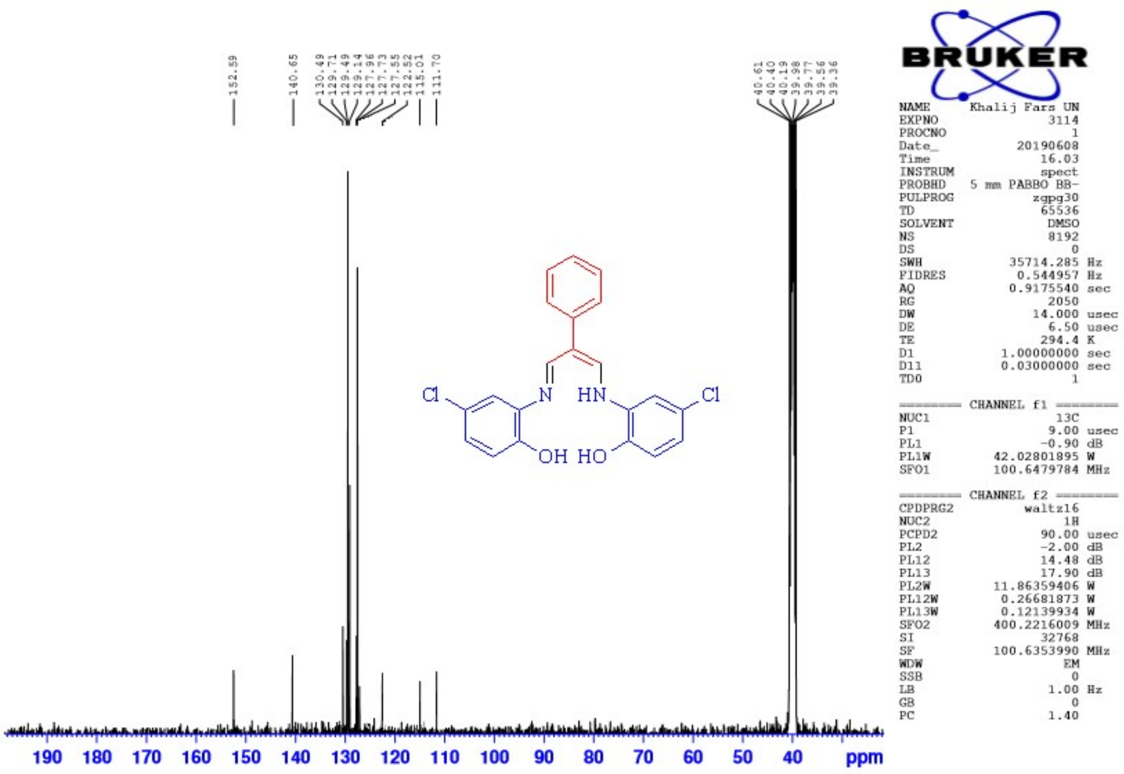

2-(4-Bromophenyl)-3-((5-chloro-2-hydroxyphenyl)amino)allylidene)amino)-4-chlorophenol 3g:

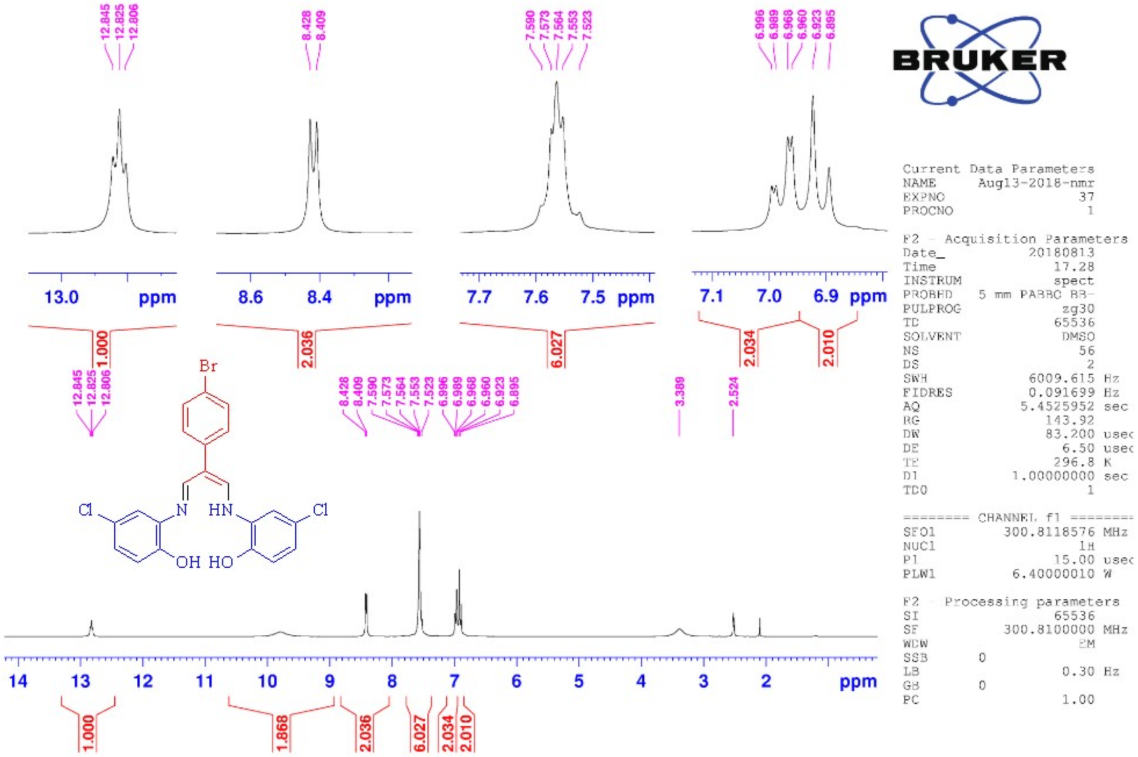

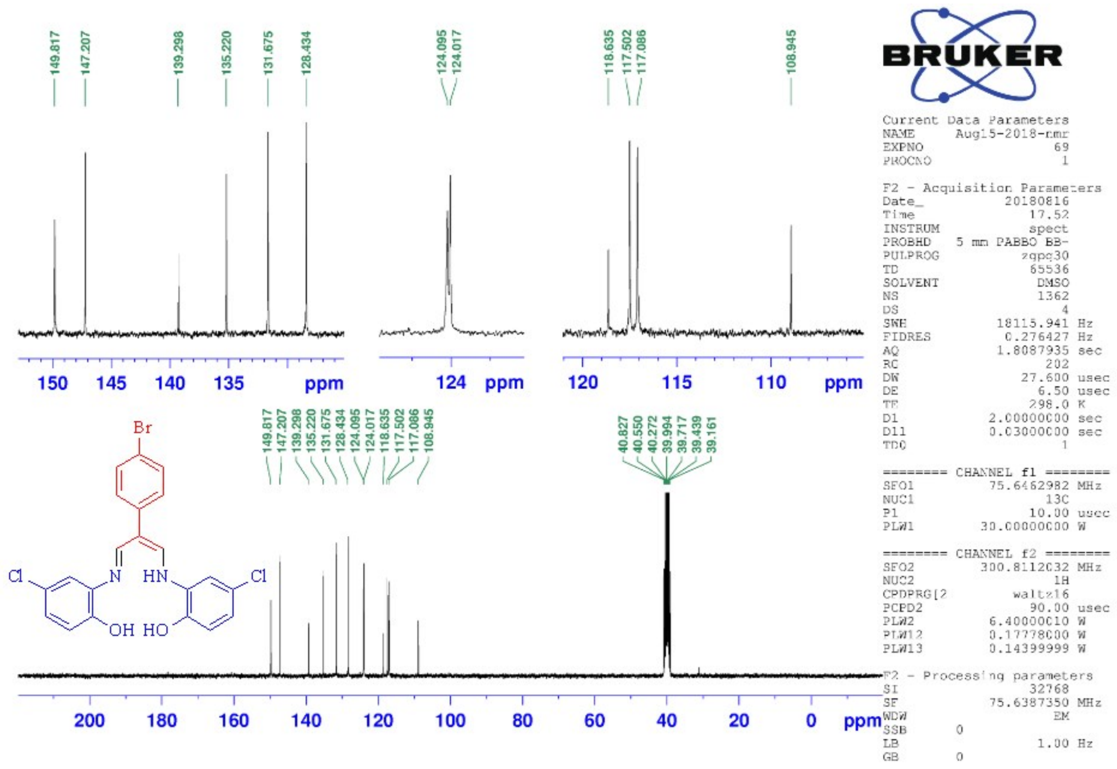

2-(4-Chlorophenyl)-3-((5-chloro-2-hydroxyphenyl)amino)allylidene)amino)-4-chlorophenol 3h:

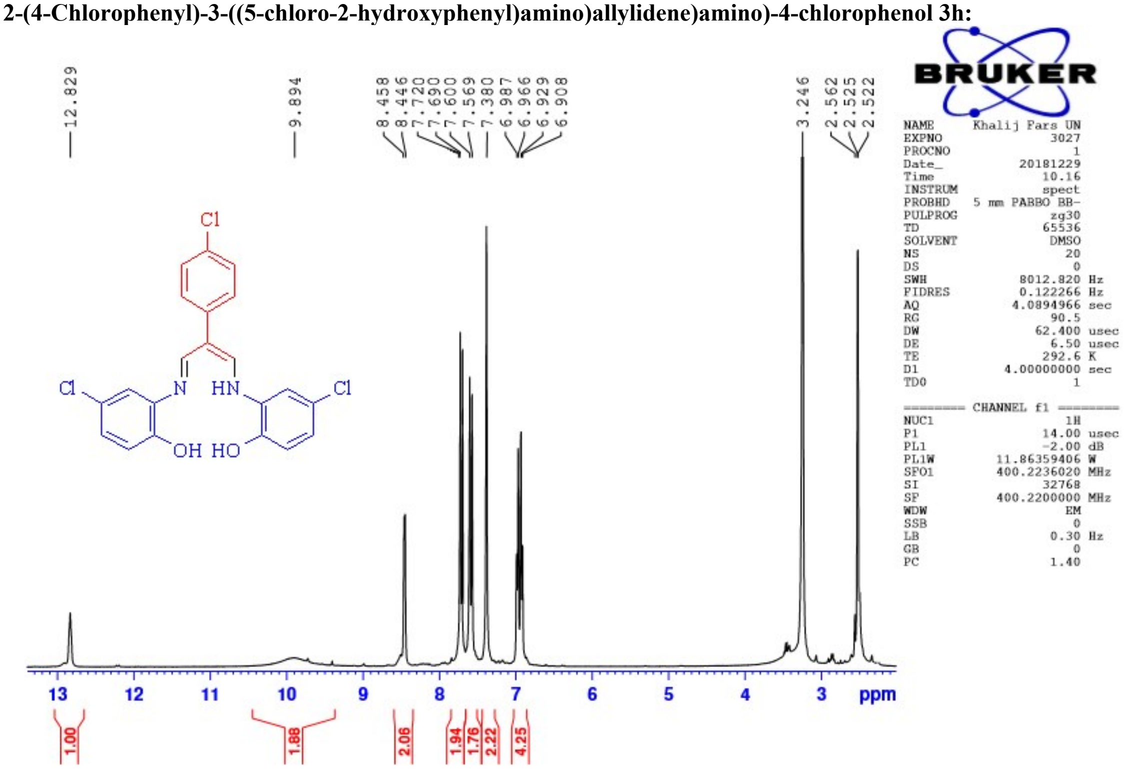

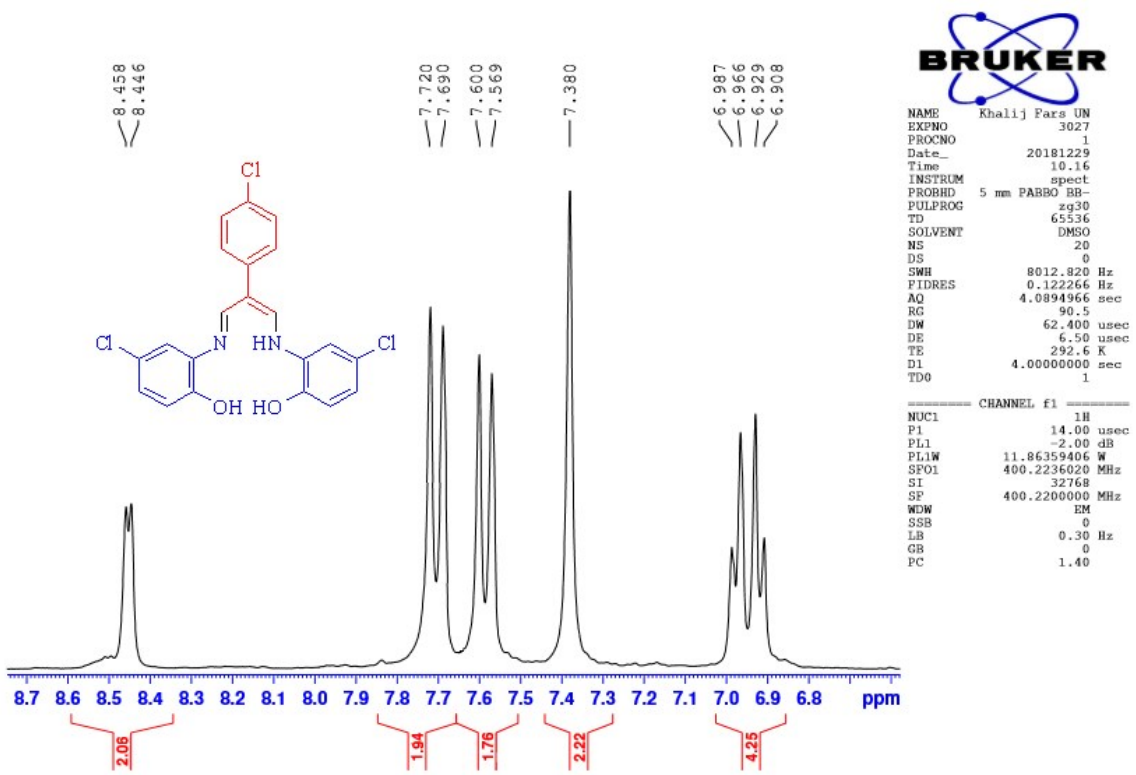

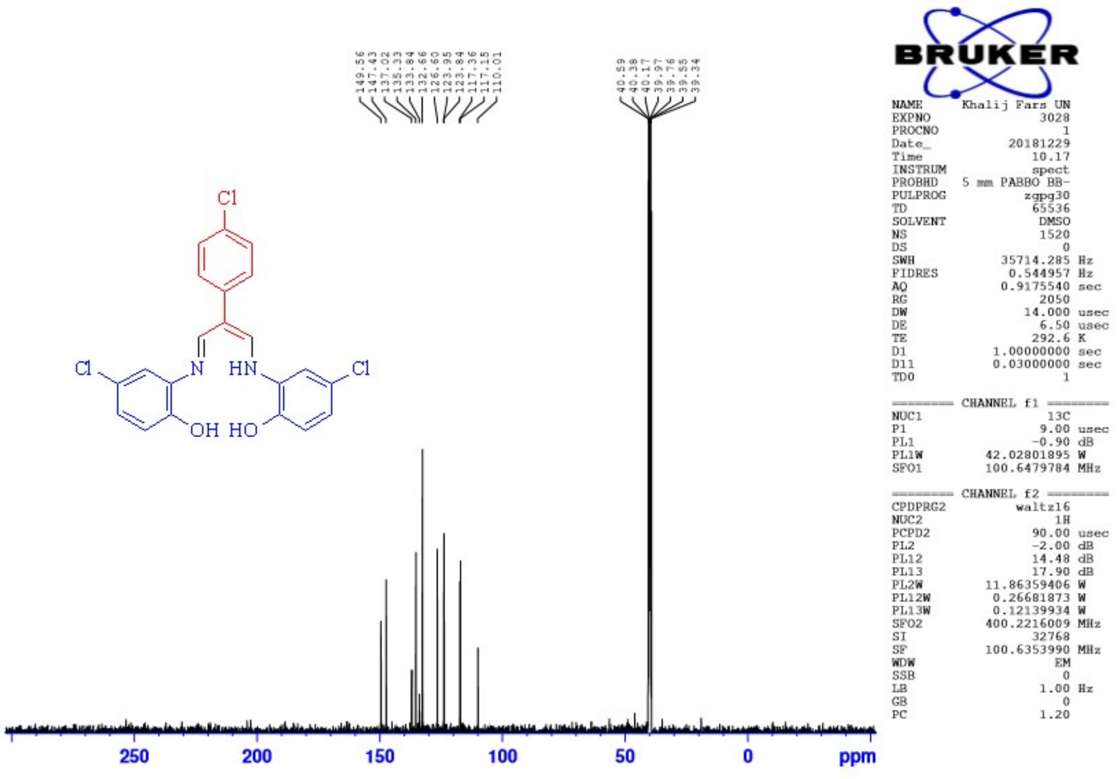

2-(4-Chlorophenyl)-3-((2-hydroxyphenyl)amino)allylidene)amino)phenol 3i:

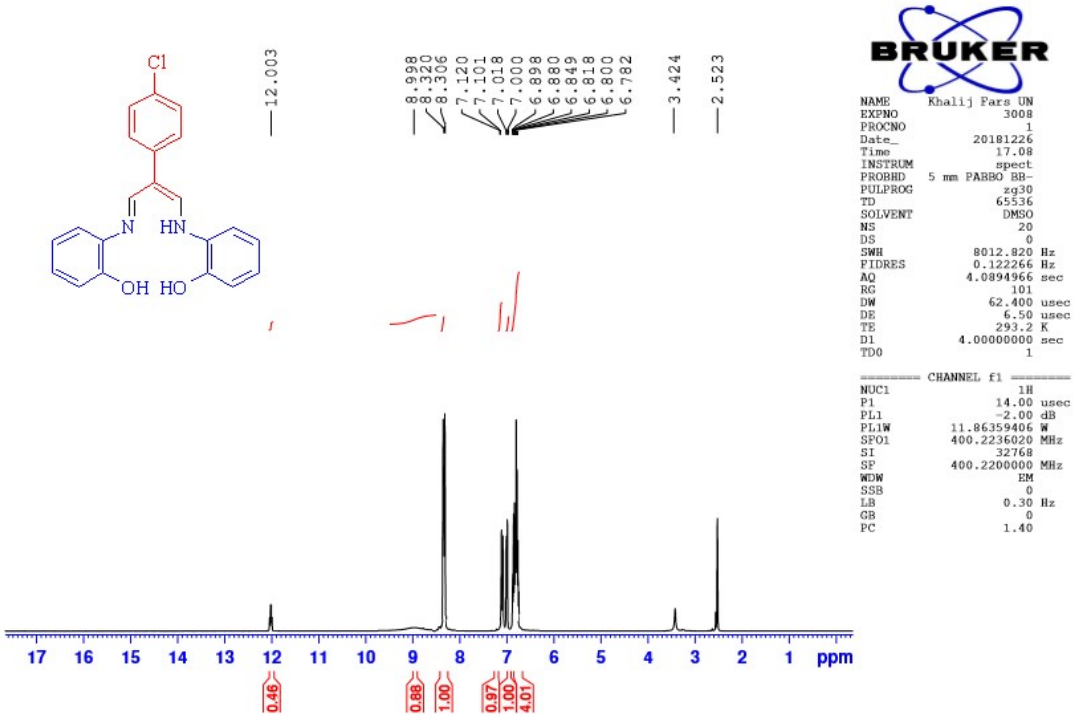
Cl
N
HN
OH HO
12.003
8.998
8.320
8.306
7.120
7.101
7.018
7.000
6.898
6.880
6.849
6.818
6.800
6.782
3.424
2.523
17 16 15 14 13 12 11 10 9 8 7 6 5 4 3 2 1 ppm
0.46
0.88
1.00
0.97
1.00
4.01
BRUKER
NAME Khalij Fars UN
EXPNO 3008
PROCNO 1
Date_ 20181226
Time 17.08
INSTRUM spect
PROBHD 5 mm PABBO BB-
PULPROG zg30
TD 65536
SOLVENT DMSO
NS 20
DS 0
SWH 8012.820 Hz
FIDRES 0.122266 Hz
AQ 4.0894966 sec
RG 101
DW 62.400 usec
DE 6.50 usec
TE 293.2 K
D1 4.00000000 sec
TD0 1
======== CHANNEL f1 ========
NUC1 1H
P1 14.00 usec
PL1 -2.00 dB
PL1W 11.86359406 W
SFO1 400.2236020 MHz
SI 32768
SF 400.2200000 MHz
WDW EM
SSB 0
LB 0.30 Hz
GB 0
PC 1.40

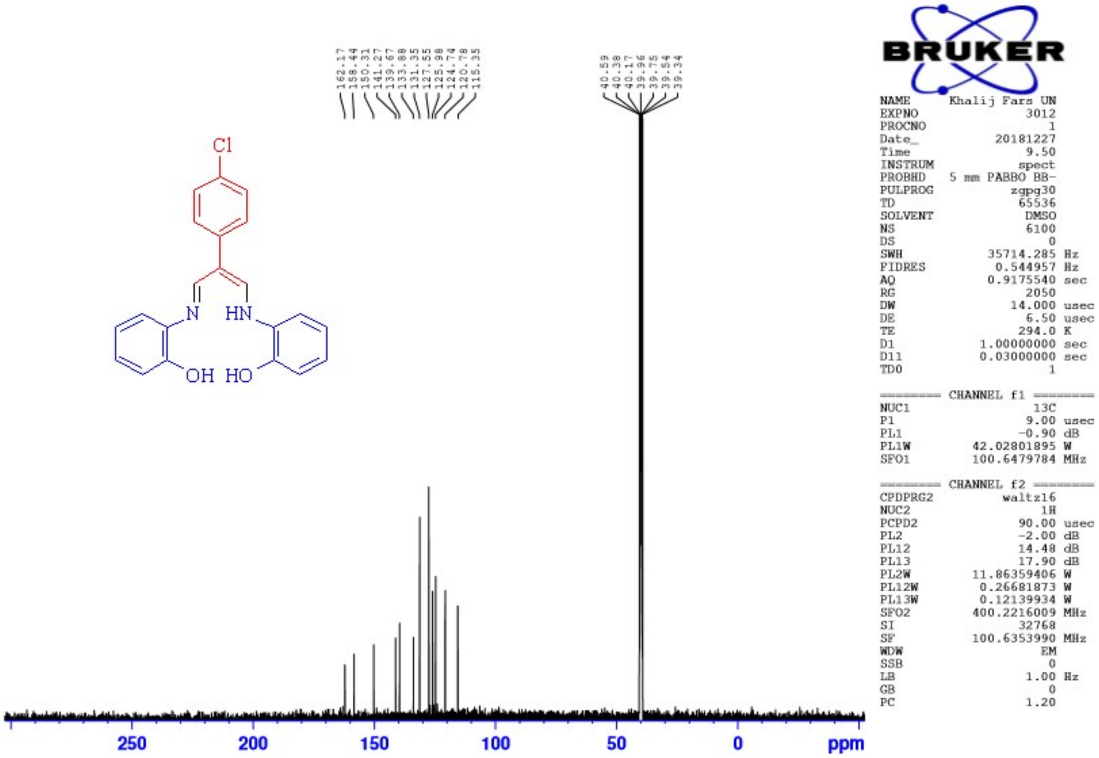
Cl
N
HN
OH HO
162.17
158.44
150.31
141.27
139.67
133.88
131.35
127.55
125.98
124.74
120.78
115.35
40.59
40.38
40.17
39.96
39.75
39.54
39.34
250 200 150 100 50 0 ppm
BRUKER
NAME Khalij Fars UN
EXPNO 3012
PROCNO 1
Date_ 20181227
Time 9.50
INSTRUM spect
PROBHD 5 mm PABBO BB-
PULPROG zgpg30
TD 65536
SOLVENT DMSO
NS 6100
DS 0
SWH 35714.285 Hz
FIDRES 0.544957 Hz
AQ 0.9175540 sec
RG 2050
DW 14.000 usec
DE 6.50 usec
TE 294.0 K
D1 1.00000000 sec
D11 0.03000000 sec
TD0 1
======== CHANNEL f1 ========
NUC1 13C
P1 9.00 usec
PL1 -0.90 dB
PL1W 42.02801895 W
SFO1 100.6479784 MHz
======== CHANNEL f2 ========
CPDPRG2 waltz16
NUC2 1H
PCPD2 90.00 usec
PL2 -2.00 dB
PL12 14.48 dB
PL13 17.90 dB
PL2W 11.86359406 W
PL12W 0.26681873 W
PL13W 0.12139934 W
SFO2 400.2216009 MHz
SI 32768
SF 100.6353990 MHz
WDW EM
SSB 0
LB 1.00 Hz
GB 0
PC 1.20

Spectral data of trimethinium salt 1c-1f:

N - (3- (dimethylamino) -2- (naphthalen-1-yl) allylidine) - N-methyl metamine perchlorate 1c:

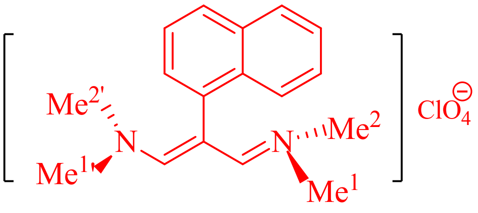

1c

Yellow powder; Yield (70%); m.p. 206-208 °C; ^{1}H NMR (DMSO-d_6, 400 MHz) δ (ppm): 2.15 (s, 6H, NMe$^{2,2'}$), 3.25 (s, 6H, NMe$^{1,1'}$), 7.48 (dd, J= 0.8, 6.8 Hz, 1H), 7.57-7.70 (m, 3H), 7.80 (d, J= 8 Hz, 2H), 7.96 (s, 2H), 8.06 (t, J= 6.6 Hz, 2H). ^{13}C NMR (DMSO-d_6, 100 MHz) δ (ppm): 49.0, 102.3, 125.7, 126.1, 127.2, 128.1, 129.1, 129.9, 130.3, 130.9, 133.2, 134.1, 164.3.

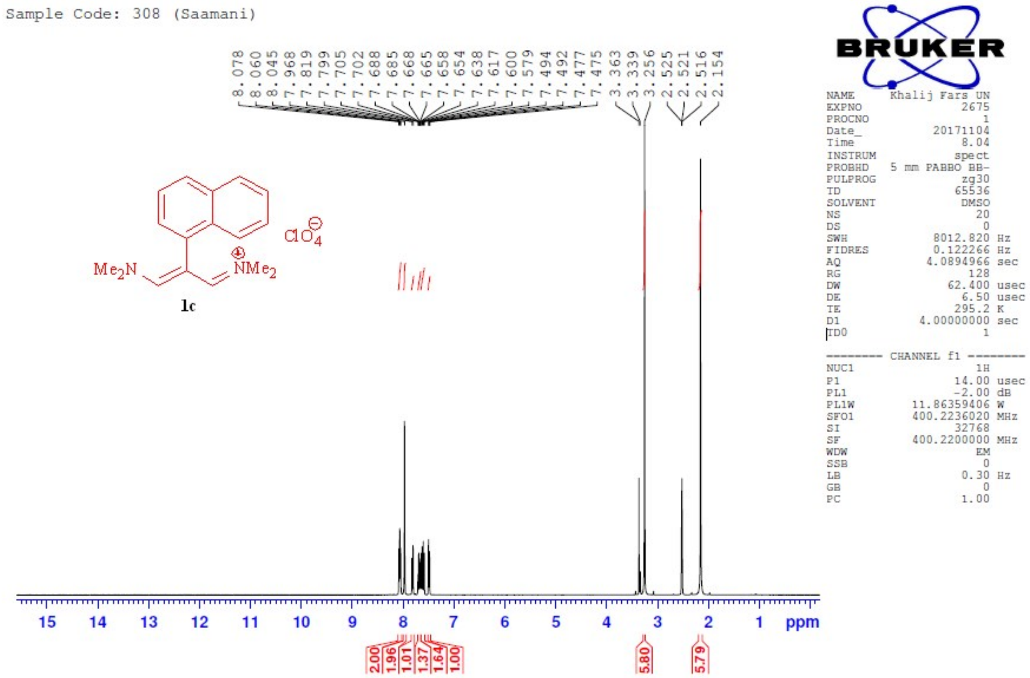

Sample Code: 308 (Saamani)

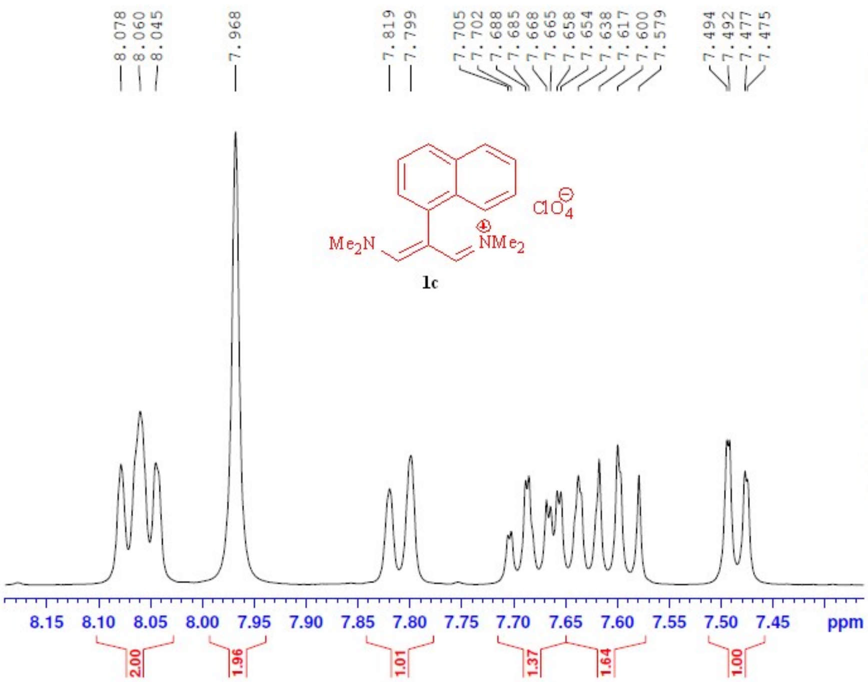

BRUKER

NAME	Khalij Fars UN
EXPNO	2675
PROCNO	1
Date_	20171104
Time	8.04
INSTRUM	spect
PROBHD	5 mm PABBO BB-
PULPROG	zg30
TD	65536
SOLVENT	DMSO
NS	20
DS	0
SWH	8012.820 Hz
FIDRES	0.122266 Hz
AQ	4.0894966 sec
RG	128
DW	62.400 usec
DE	6.50 usec
TE	295.2 K
D1	4.00000000 sec
TD0	1

======== CHANNEL f1 ========

NUC1	1H
P1	14.00 usec
PL1	-2.00 dB
PL1W	11.86359406 W
SFO1	400.2236020 MHz
SI	32768
SF	400.2200000 MHz
WDW	EM
SSB	0
LB	0.30 Hz
GB	0
PC	1.00

Sample Code:308 (Saamani)

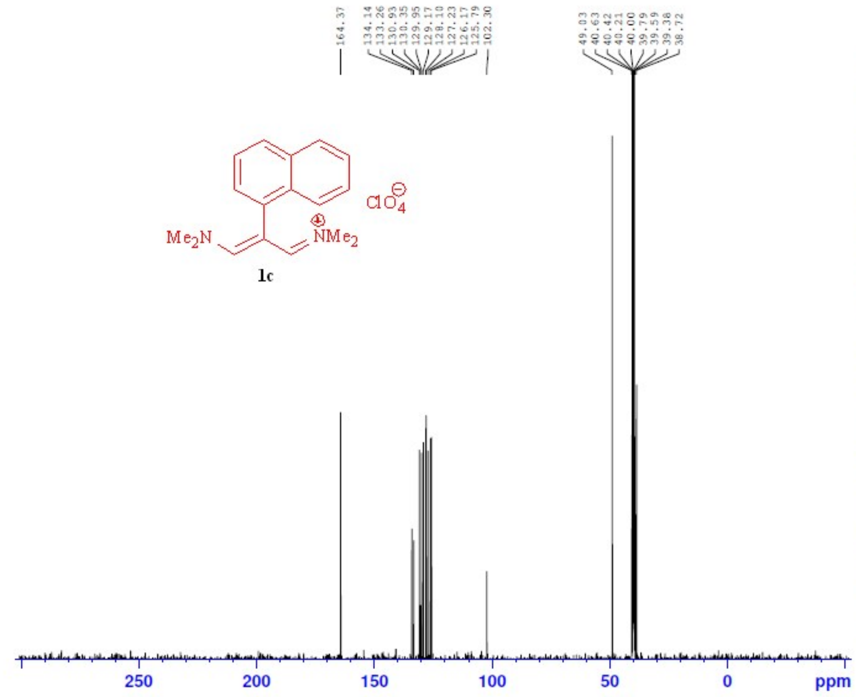

BRUKER

NAME	Khalij Fars UN
EXPNO	2732
PROCNO	1
Date_	20171108
Time	17.48
INSTRUM	spect
PROBHD	5 mm PABBO BB-
PULPROG	zgpg30
TD	65536
SOLVENT	DMSO
NS	476
DS	0
SWH	35714.285 Hz
FIDRES	0.544957 Hz
AQ	0.9175540 sec
RG	50.8
DW	14.000 usec
DE	6.50 usec
TE	295.9 K
D1	1.00000000 sec
D11	0.03000000 sec
TD0	1

======== CHANNEL f1 ========

NUC1	13C
P1	9.00 usec
PL1	-0.90 dB
PL1W	42.02801895 W
SFO1	100.6479784 MHz

======== CHANNEL f2 ========

CPDPRG2	waltz16
NUC2	1H
PCPD2	90.00 usec
PL2	-2.00 dB
PL12	14.48 dB
PL13	17.90 dB
PL2W	11.86359406 W
PL12W	0.26681873 W
PL13W	0.12139934 W
SFO2	400.2216009 MHz
SI	32768
SF	100.6353990 MHz
WDW	EM
SSB	0
LB	1.00 Hz
GB	0
PC	1.40

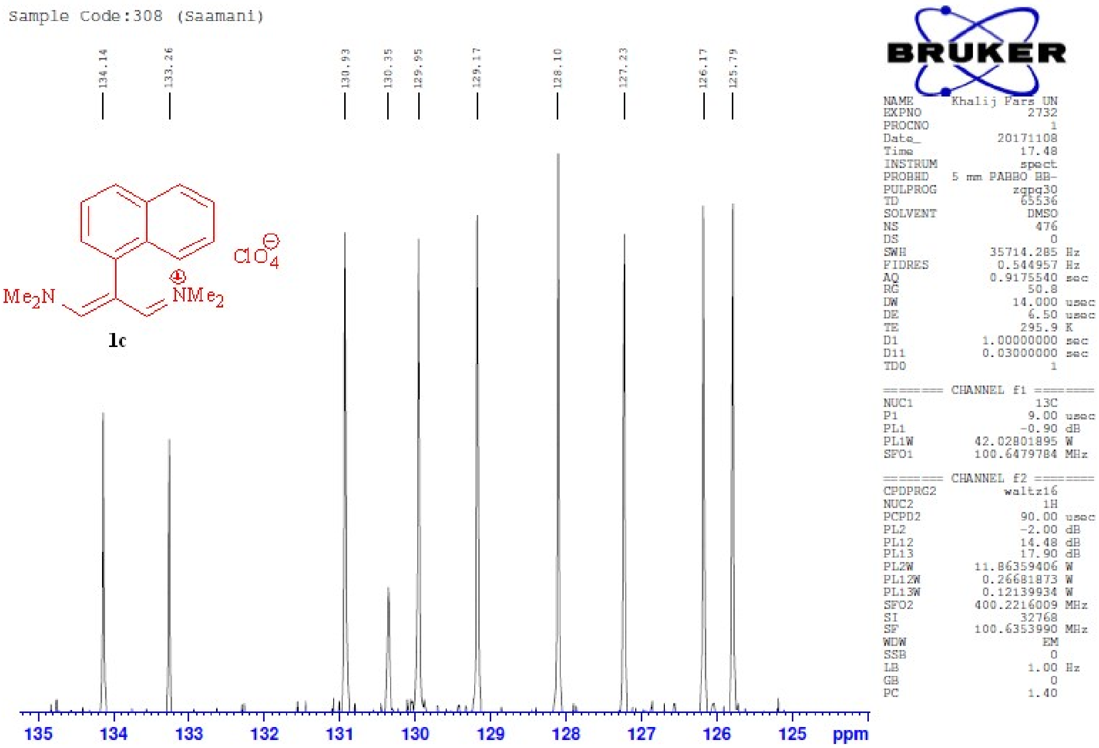

N - (3- (dimethylamino) -2- (phenylallylidine) - N-methyl metamine perchlorate 1d:

White powder; Yield (75%); m.p. 200-202 °C; ^{1}H NMR (DMSO-d_6, 400 MHz) δ (ppm): 2.43 (s, 6H), 3.25 (s, 6H), 7.31-7.33 (m, 2H), 7.45 (t, *J*= 3 Hz, 3H), 7.72 (s, 2H). ^{13}C NMR (DMSO-d_6, 100 MHz) δ (ppm): 49.0, 105.5, 128.9, 129.2, 132.6, 133.0, 163.3.

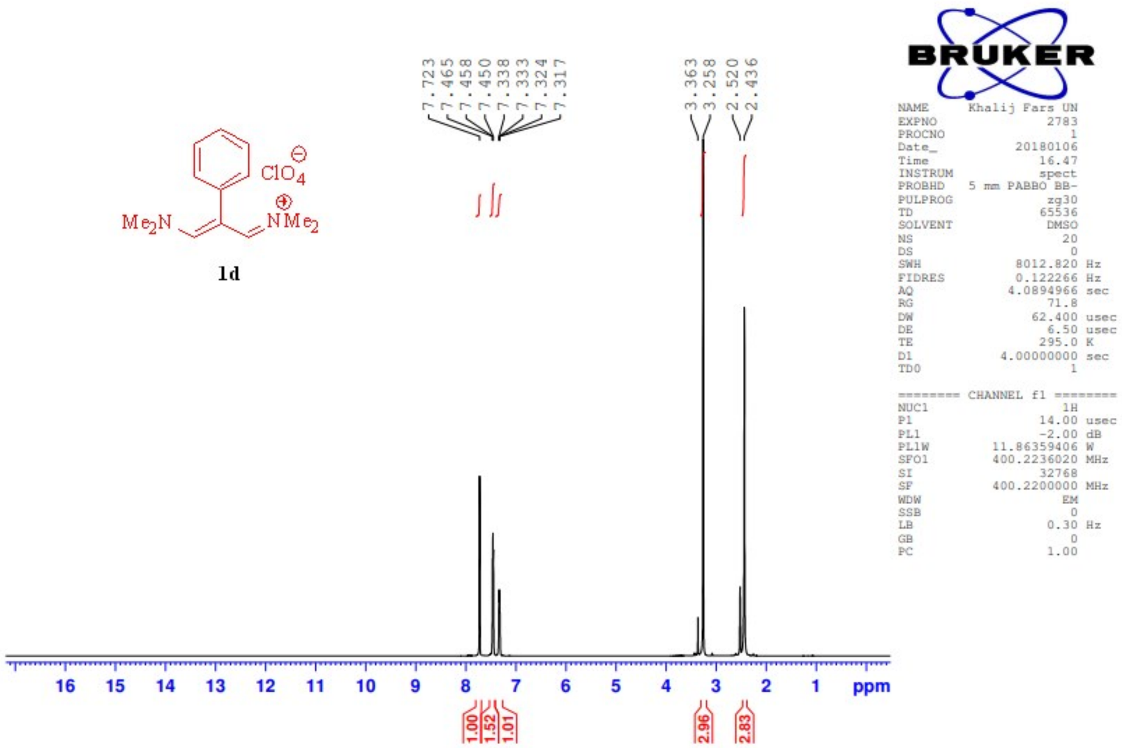
7.723
7.465
7.458
7.450
7.338
7.333
7.324
7.317
3.363
3.258
2.520
2.436
ClO4
Me2N
NMe2
1d
16 15 14 13 12 11 10 9 8 7 6 5 4 3 2 1 ppm
1.00
1.52
1.01
2.96
2.83
BRUKER
NAME Khalij Fars UN
EXPNO 2783
PROCNO 1
Date_ 20180106
Time 16.47
INSTRUM spect
PROBHD 5 mm PABBO BB-
PULPROG zg30
TD 65536
SOLVENT DMSO
NS 20
DS 0
SWH 8012.820 Hz
FIDRES 0.122266 Hz
AQ 4.0894966 sec
RG 71.8
DW 62.400 usec
DE 6.50 usec
TE 295.0 K
D1 4.00000000 sec
TD0 1
======== CHANNEL f1 ========
NUC1 1H
P1 14.00 usec
PL1 -2.00 dB
PL1W 11.86359406 W
SFO1 400.2236020 MHz
SI 32768
SF 400.2200000 MHz
WDW EM
SSB 0
LB 0.30 Hz
GB 0
PC 1.00

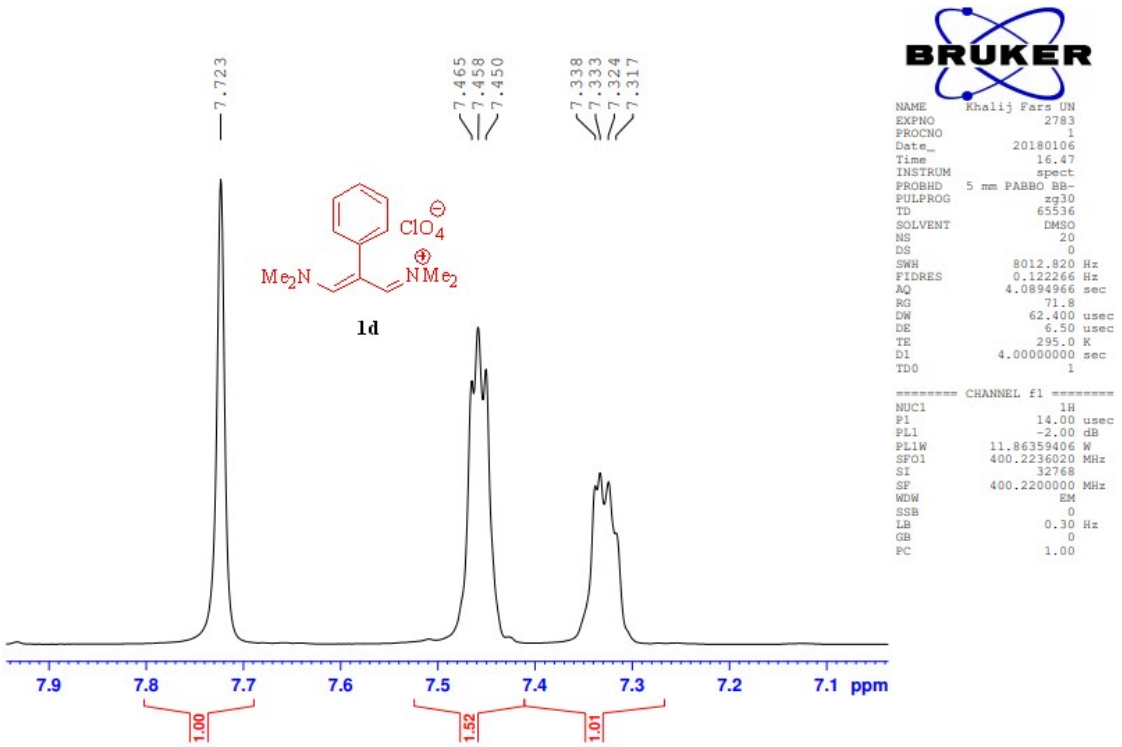
7.723
7.465
7.458
7.450
7.338
7.333
7.324
7.317
ClO4
Me2N
NMe2
1d
7.9 7.8 7.7 7.6 7.5 7.4 7.3 7.2 7.1 ppm
1.00
1.52
1.01
BRUKER
NAME Khalij Fars UN
EXPNO 2783
PROCNO 1
Date_ 20180106
Time 16.47
INSTRUM spect
PROBHD 5 mm PABBO BB-
PULPROG zg30
TD 65536
SOLVENT DMSO
NS 20
DS 0
SWH 8012.820 Hz
FIDRES 0.122266 Hz
AQ 4.0894966 sec
RG 71.8
DW 62.400 usec
DE 6.50 usec
TE 295.0 K
D1 4.00000000 sec
TD0 1
======== CHANNEL f1 ========
NUC1 1H
P1 14.00 usec
PL1 -2.00 dB
PL1W 11.86359406 W
SFO1 400.2236020 MHz
SI 32768
SF 400.2200000 MHz
WDW EM
SSB 0
LB 0.30 Hz
GB 0
PC 1.00

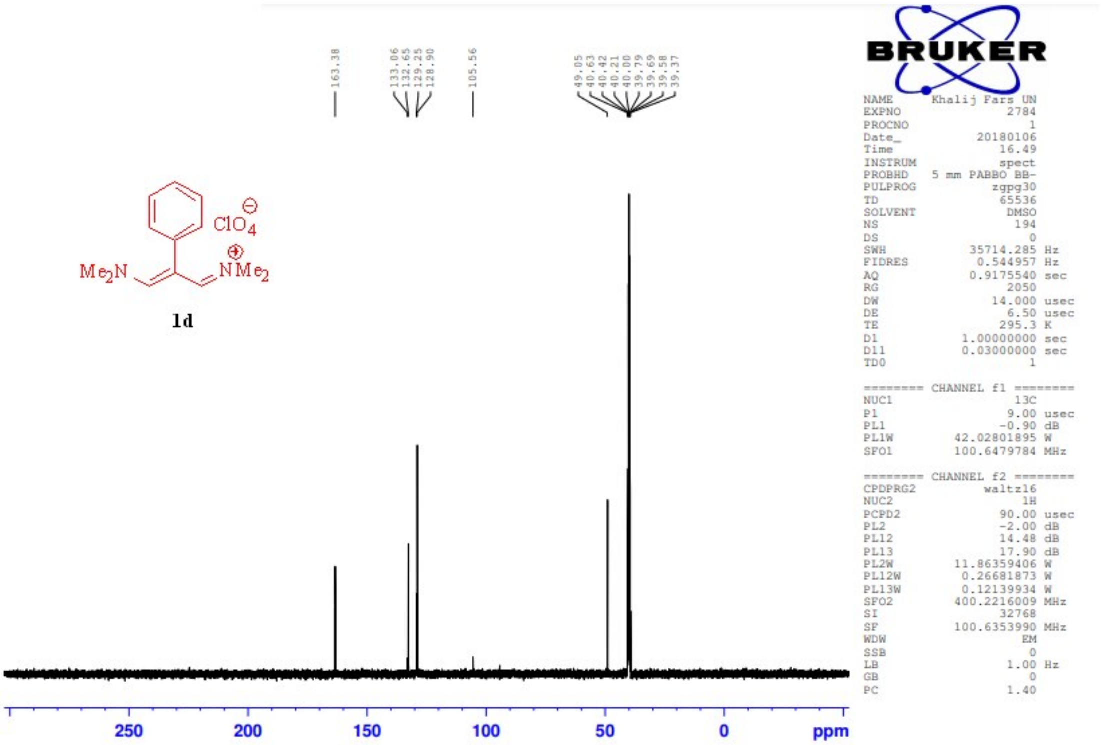

N-(2-(4-bromophenyl)-3-(dimethylamino) -allylidene)-N-methylmethanaminium perchlorate 1e:

White powder; Yield (85%); m.p. 148-150 °C; ^{1}H NMR (DMSO-d_6, 300 MHz) δ (ppm): 2.47 (s, 6H), 3.26 (s, 6H), 7.29 (d, *J*= 8.1 Hz, 2H), 7.64 (d, *J*= 8.4 Hz, 2H), 7.72 (s, 2H). ^{13}C NMR (DMSO-d_6, 75 MHz) δ (ppm): 49.1, 104.0, 122.6, 131.7, 132.4, 134.7, 163.3.

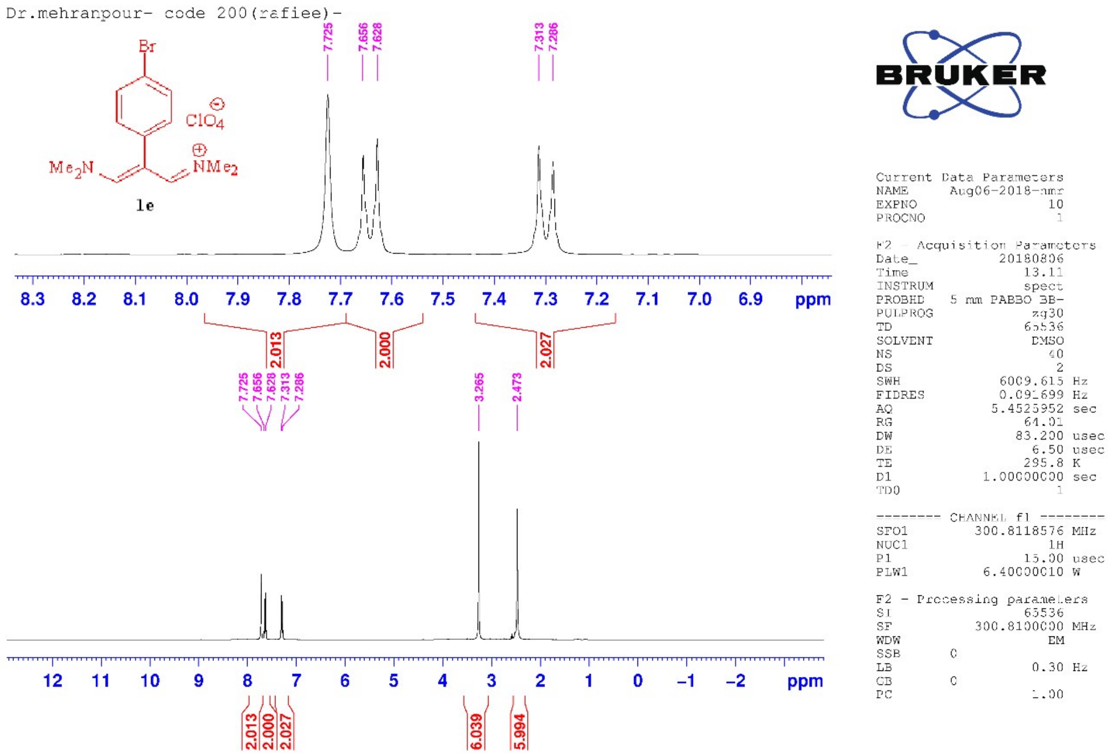
Dr.mehranpour- code 200(rafiee)-
Br
ClO4
NMe2
Me2N
1e
7.725
7.656
7.628
7.313
7.286
2.013
2.000
2.027
3.265
2.473
6.039
5.994
ppm
BRUKER
Current Data Parameters
NAME Aug06-2018-nmr
EXPNO 10
PROCNO 1
F2 - Acquisition Parameters
Date_ 20180806
Time 13.11
INSTRUM spect
PROBHD 5 mm PABBO BB-
PULPROG zg30
TD 65536
SOLVENT DMSO
NS 40
DS 2
SWH 6009.615 Hz
FIDRES 0.091699 Hz
AQ 5.4525952 sec
RG 64.01
DW 83.200 usec
DE 6.50 usec
TE 295.8 K
D1 1.00000000 sec
TD0 1
======== CHANNEL f1 ========
SFO1 300.8118576 MHz
NUC1 1H
P1 15.00 usec
PLW1 6.40000010 W
F2 - Processing parameters
SI 65536
SF 300.8100000 MHz
WDW EM
SSB 0
LB 0.30 Hz
GB 0
PC 1.00

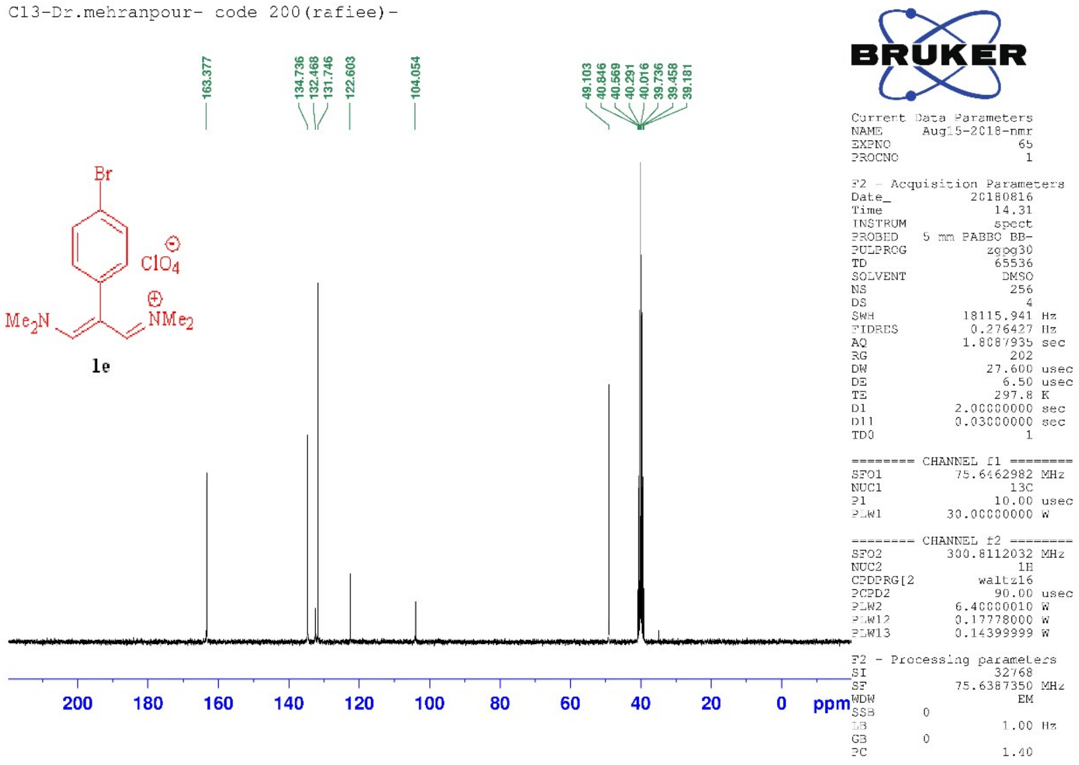
C13-Dr.mehranpour- code 200(rafiee)-
163.377
134.736
132.468
131.746
122.603
104.054
49.103
40.846
40.569
40.291
40.016
39.736
39.458
39.181
Br
ClO4
NMe2
Me2N
1e
ppm
BRUKER
Current Data Parameters
NAME Aug15-2018-nmr
EXPNO 65
PROCNO 1
F2 - Acquisition Parameters
Date_ 20180816
Time 14.31
INSTRUM spect
PROBHD 5 mm PABBO BB-
PULPROG zgpg30
TD 65536
SOLVENT DMSO
NS 256
DS 4
SWH 18115.941 Hz
FIDRES 0.276427 Hz
AQ 1.8087935 sec
RG 202
DW 27.600 usec
DE 6.50 usec
TE 297.8 K
D1 2.00000000 sec
D11 0.03000000 sec
TD0 1
======== CHANNEL f1 ========
SFO1 75.6462982 MHz
NUC1 13C
P1 10.00 usec
PLW1 30.00000000 W
======== CHANNEL f2 ========
SFO2 300.8112032 MHz
NUC2 1H
CPDPRG[2 waltz16
PCPD2 90.00 usec
PLW2 6.40000010 W
PLW12 0.17778000 W
PLW13 0.14399999 W
F2 - Processing parameters
SI 32768
SF 75.6387350 MHz
WDW EM
SSB 0
LB 1.00 Hz
GB 0
PC 1.40

N-(2-(4-chlorophenyl)-3-(dimethylamino) -allylidene)-N-methylmethanaminium perchlorate 1f:

White powder; Yield (80%); m.p. 89-91 °C; ^{1}H NMR (DMSO-d_6, 300 MHz) δ (ppm): 2.46 (s, 6H), 3.16 (s, 6H), 7.36 (d, *J*= 8.1 Hz, 2H), 7.49 (d, *J*= 8.4 Hz, 2H), 7.71 (s, 2H). ^{13}C NMR (DMSO-d_6, 75 MHz) δ (ppm): 50.0, 105.0, 121.3, 129.3, 132.1, 134.6, 163.5.

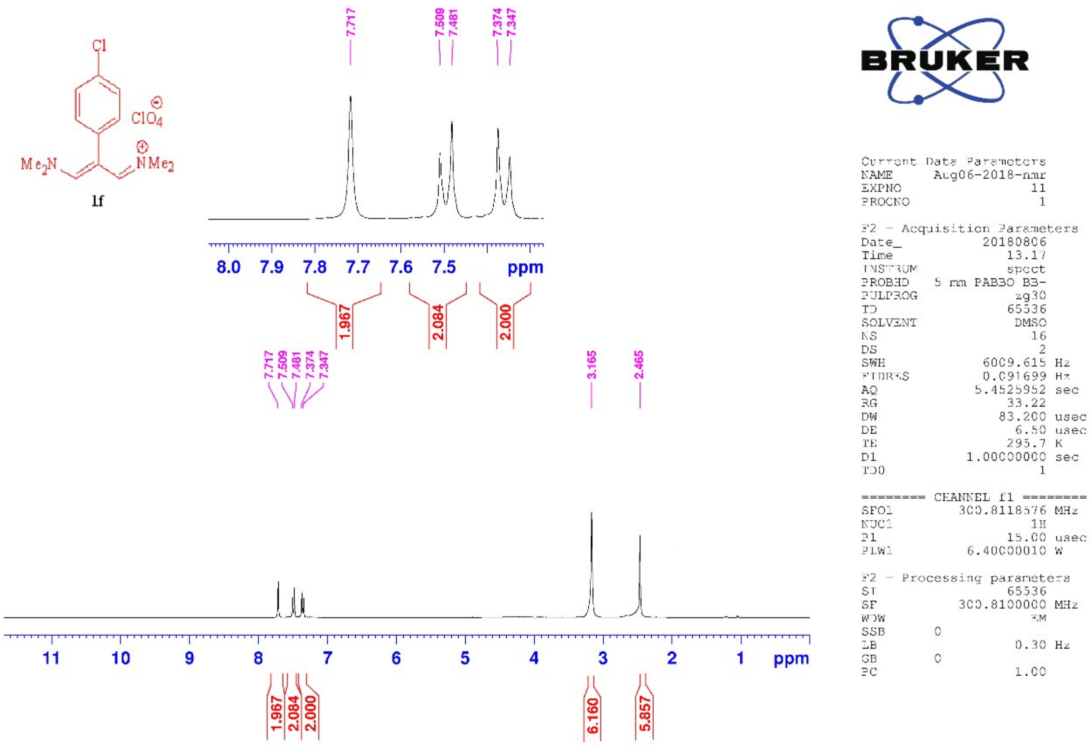

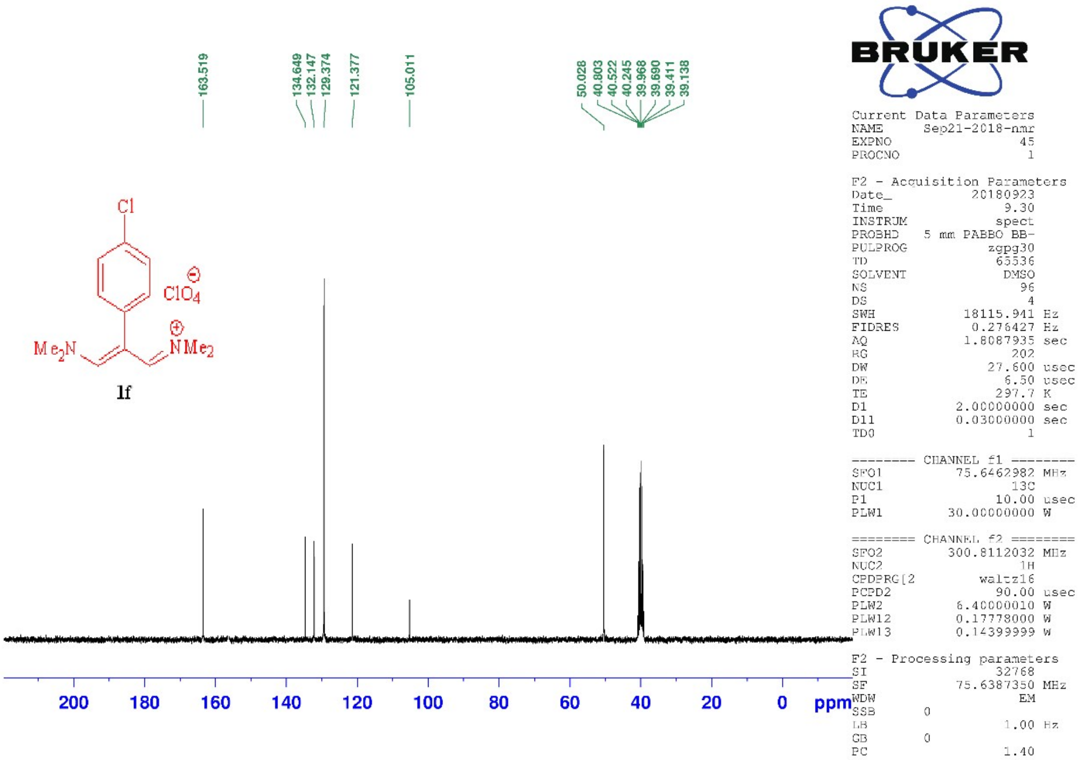
163.519
134.649
132.147
129.374
121.377
105.011
50.028
40.803
40.522
40.245
39.968
39.690
39.411
39.138
Cl
ClO_4
Me_2N
NMe_2
1f
200
180
160
140
120
100
80
60
40
20
0
ppm
BRUKER
Current Data Parameters
NAME Sep21-2018-nmr
EXPNO 45
PROCNO 1
F2 - Acquisition Parameters
Date_ 20180923
Time 9.30
INSTRUM spect
PROBHD 5 mm PABBO BB-
PULPROG zgpg30
TD 65536
SOLVENT DMSO
NS 96
DS 4
SWH 18115.941 Hz
FIDRES 0.276427 Hz
AQ 1.8087935 sec
RG 202
DW 27.600 usec
DE 6.50 usec
TE 297.7 K
D1 2.00000000 sec
D11 0.03000000 sec
TD0 1
======== CHANNEL f1 ========
SFO1 75.6462982 MHz
NUC1 13C
P1 10.00 usec
PLW1 30.00000000 W
======== CHANNEL f2 ========
SFO2 300.8112032 MHz
NUC2 1H
CPDPRG[2 waltz16
PCPD2 90.00 usec
PLW2 6.40000010 W
PLW12 0.17778000 W
PLW13 0.14399999 W
F2 - Processing parameters
SI 32768
SF 75.6387350 MHz
WDW EM
SSB 0
LB 1.00 Hz
GB 0
PC 1.40
